# Comprehensive analysis of the autophagy-dependent ferroptosis-related gene FANCD2 in lung adenocarcinoma

**DOI:** 10.1186/s12885-022-09314-9

**Published:** 2022-03-02

**Authors:** Huikai Miao, Qiannan Ren, Hongmu Li, Mingyue Zeng, Dongni Chen, Chunmei Xu, Youfang Chen, Zhesheng Wen

**Affiliations:** 1 Department of Thoracic Oncology, State Key Laboratory of Oncology in South China, Collaborative Innovation Center for Cancer Medicine, Sun Yat-sen University Cancer Center, 651 Dongfengdong, Guangzhou, 510060 People’s Republic of China; 2 Department of Nasopharyngeal Carcinoma, State Key Laboratory of Oncology in South China, Collaborative Innovation Center for Cancer Medicine, Sun Yat-sen University Cancer Center, Guangzhou, 510060 People’s Republic of China; 3 Department of Endocrinology, Shandong Provincial Hospital Affiliated to Shandong First Medical University, Jinan, 250021 People’s Republic of China

**Keywords:** Lung adenocarcinoma, Autophagy-dependent ferroptosis, FANCD2, Prognosis, Immunity

## Abstract

**Background:**

The development of lung adenocarcinoma (LUAD) involves the interactions between cell proliferation and death. Autophagy-dependent ferroptosis, a distinctive cell death process, was implicated in a multitude of diseases, whereas no research revealing the relationship between autophagy-dependent ferroptosis and LUAD pathogenesis was reported. Thus, the primary objective was to explore the role and potential function of the autophagy-dependent ferroptosis-related genes in LUAD.

**Methods:**

Clinical information and transcriptome profiling of patients with LUAD were retrieved and downloaded from open-source databases. Autophagy-dependent ferroptosis-related genes were screened by published articles. The critical gene was identified as the intersection between the differentially expressed genes and prognosis-related genes. Patients were divided into high- and low-risk groups using the expression level of the critical gene. The validity of the key gene prognosis model was verified by survival analysis. The correlation between the clinical characteristics of LUAD and the expression level of the key gene was analyzed to explore the clinical significance and prognosis value. And the roles of the key gene in response to chemotherapy, immune microenvironment, and tumor mutation burden were predicted. The validation of key gene expression levels was further performed by quantitative real-time PCR and immunohistochemistry staining.

**Results:**

*FANCD2*, an essential autophagy-dependent ferroptosis-related gene by searching database, was confirmed as an independent prognostic factor for LUAD occurrence. The high expression level of *FANCD2* was associated with an advantaged TNM stage, a less chemotherapy sensitivity, a low ImmuneScore, which indicated a deactivation status in an immune microenvironment, a high tumor mutation burden, and poor survival for LUAD patients. Pathway enrichment analysis showed that *FANCD2* responded to oxidative stress and neutrophil-mediated immunity. Quantitative real-time PCR and immunohistochemistry staining showed that the expression level of *FANCD2* is higher in LUAD patients than in normal tissue samples, which was in accordance with the database report.

**Conclusion:**

*FANCD2*, an essential gene related to autophagy-dependent ferroptosis, could work as a biomarker, predicting the survival, chemotherapy sensitivity, tumor immunity, and mutation burden of LUAD. Researching autophagy-dependent ferroptosis and targeting the *FANCD2* may offer a new perspective for treating and improving prognosis in LUAD.

**Supplementary Information:**

The online version contains supplementary material available at 10.1186/s12885-022-09314-9.

## Background

Lung adenocarcinoma (LUAD) is one of the most common malignant tumors in the world, demonstrating a rising trend in recent years [1]. Due to the high recurrence and metastasis, traditional treatments, such as surgery, radiotherapy, and chemotherapy, could not meet all LUAD patients’ needs. Although immunotherapy has been shown to improve survival in LUAD patients, the 5-year overall survival rate is only 23% [2]. The pathogenic mechanism of LUAD should be further elucidated to discover a new effective treatment strategy.

The tumor heterogeneity, including immune microenvironment and tumor mutation burden, could affect immunotherapy effectiveness. Ferroptosis is also involved in T cell immunity and cancer immunotherapy. The increased ferroptosis contributes to the anti-tumor efficacy of immunotherapy [3].

Ferroptosis is an iron-dependent form of regulated cell death that is characterized by the excess reactive oxygen species (ROS) generation and lethal accumulation of lipid peroxidation [4–6]. Ferroptosis has been implicated in multiple physiological and pathological processes, including cancer cell death and T-cell immunity [7]. Autophagy-dependent ferroptosis is featured by excessive autophagy and lysosome activity [8]. The influence of ferroptosis, especially autophagy-dependent ferroptosis, on the tumor microenvironment needs further study.

The iron metabolism and homeostasis could be influenced by immune cells and related molecules [9]. Immune cells in the microenvironment play crucial roles in maintaining iron metabolism balance [10]. The excessive activation of ferroptosis in tumor cells can lead to exposure to tumor antigens, which activate the immune system. Then, the immunogenicity of the microenvironment was improved, and the effectiveness of immunotherapy was enhanced [11]. Immunotherapy can activate CD8 + T cells to enhance the lipid peroxidation in tumor cells, which further increases ferroptosis in turn [3]. Therefore, targeting ferroptosis to improve the effectiveness of cancer immunotherapy might become a prospective strategy. In the clinical applications of immunotherapy, tumor mutation burden (TMB) is emphasized as an emerging feature and a biomarker of immunotherapy response [12, 13]. TMB is defined as the total number of somatic, coding, base substitution, and indel mutations per megabase of genome examined [14]. Each of these mutations results in the generation of one protein that is a new antigen and could be recognized by the immune system [15]. Highly mutated tumors are more likely to carry neoantigens, making them become the targets for activated immune cells [14].

In this study, we comprehensively analyze the genome of LUAD, identify autophagy-dependent ferroptosis-related genes closely associated with the prognosis and chemotherapy sensitivity, further construct and validate the predictive model of the key gene, and explore the relationship with immune infiltration and tumor mutation. Our findings may help generate personalized treatment and improve the clinical outcomes of LUAD patients.

## Materials and methods

### Workflow

A multi-step approach was used to identify and analyze the autophagy-dependent ferroptosis-related key gene in LUAD. The transcriptome and clinical information were downloaded from The Cancer Genome Atlas (TCGA) project and Gene Expression Omnibus (GEO) data. Autophagy-dependent ferroptosis-related genes were screened by the published articles. Differentially expressed genes (DEGs) related to autophagy-dependent ferroptosis were identified. Univariate and multivariate Cox analyses were applied to screen out the independent prognosis genes related to overall survival (OS). The key gene was identified by the intersection of the DEGs and the prognostic genes. The LUAD patients were classified into the high-risk and low-risk groups based on the key gene expression level. Kaplan-Meier (K-M) analysis and receiver operating characteristic (ROC) curve were conducted to analyze the survival prognosis of patients in TCGA and GEO cohorts. Chemotherapy sensitivity was predicted between different risk groups. Gene ontology (GO) and Kyoto encyclopedia of genes and genomes (KEGG) were conducted to investigate the potential bio-function of the key gene. ImmuneScore was calculated using the Tumor Immune Estimation Resource (TIMER) algorithm, and the TMB was counted as the total number of mutations per megabyte of tumor tissue.

### LUAD patients dataset processing

All the RNA-Seq data were normalized as fragments per kilobase of transcript per million mapped reads. mRNAs ensemble gene identities were derived from the HUGO Gene Nomenclature Committee (HGNC) database. The corresponding clinical information includes age, gender, tumor grade, lymph node metastasis, AJCC TNM stages, and survival outcomes. Patients with insufficient clinical data were excluded. OS was estimated as the primary endpoint.

### Construction and validation of an autophagy-dependent ferroptosis-related gene signature

Autophagy-dependent ferroptosis-related genes were retrieved from the literature published before January 2021. After combining the related mRNA expression and the clinical data, the gene expression files were obtained. The DEGs between LUAD and normal lung tissues were identified with a false discovery rate (FDR) < 0.05 in the TCGA cohort. Univariate and multivariate Cox analyses of OS were performed to screen the genes with prognostic values in TCGA-LUAD cohort. The key gene was identified by the intersection of the DEGs and the prognostic genes in the TCGA cohort. The cut-off score was defined as the median expression level of the key gene in the LUAD cohort. Patients were stratified into high-risk and low-risk groups based on the cut-off score. To choose appropriate matching cohorts to perform survival analysis before selecting prognostic-related genes, we performed propensity score matching to reduce the selection bias between the high- and low-risk groups. Propensity scores were estimated using age, gender (male versus female), TNM stage (I, II, III, IV), Tumor stage (T1, T2, T3, T4), Lymph Node stage (N0, N1, N2, N3) and Metastasis stage (M0, M1) in TCGA-LUAD cohort. In the same way, propensity scores were estimated using age, gender (male versus female), TNM stage (I, II, III, IV), and *TP53* (Wild versus Mutant) in the GEO cohort. The prognostic value and the clinical correlation of the key gene were both validated between the high- and low-risk groups in TCGA and GEO cohort (GSE116959). The time-dependent ROC curve analyses were conducted to evaluate the predictive power of the key gene. The mRNA expression level of the key gene in various types of cancers was identified in the Oncomine database [16]. The mRNA and protein expression of the key gene in LUAD were determined using the Gene Expression Profiling Interactive Analysis (GEPIA) and The Human Protein Atlas (HPA) database [17, 18]. To verify the correlation between ferroptosis and LUAD outcome, we also analyze the survival value of *GPX4* in the LUAD cohort, which is the master regulator of ferroptosis [19].

### Chemotherapeutic response prediction

We analyzed the commonly used chemotherapy drugs, including pemetrexed, cisplatin, gemcitabine, paclitaxel, vinorelbine, docetaxel, doxorubicin, etoposide, erlotinib, and gefitinib [20]. The chemotherapeutic response prediction was made based on the TCGA-LUAD cohort using the “pRRophetic” R package [21]. The half maximal inhibitory concentration (IC50) of patients in different risk groups were compared.

### Functional enrichment analysis

The biological functions and pathways of the key gene were elucidated through the DEGs between the high-risk and low-risk groups. GO enrichment and KEGG pathway analyses [22] were then assessed in DAVID database. The correlation analysis of the key gene with tumor proliferation and cell cycle markers was conducted in GEPIA database [17].

### Correlation between the key gene and tumor immune cell infiltration

The enrichment levels of immune cells were quantified by the Tumor Purity, Estimate Score, Immune Score, and Stromal Score in each sample. The tumor immune cell infiltration was calculated by Single Sample Gene Set Enrichment Analysis (ssGSEA). Then we analyzed the correlation between the key gene expression and the abundance of infiltrating immune cells (B cells, CD8+ T cells, CD4+ T cells, macrophages, neutrophils, and dendritic cells) via The Tumor IMmune Estimation Resource (TIMER) database [23].

### Analyses of somatic mutations and TMB estimation

The somatic mutation profiles of LUAD patients were downloaded from TCGA database. The mutation frequency with the number of variants/the length of exons (38 million) were calculated for each sample. The OncoPlot of the top 10 mutated genes was plotted. The detailed mutational information, including the variant classification, the number of variant type, and the single-nucleotide variant (SNV) class, were displayed. Then we assessed the correlation between the key gene expression and the TMB levels.

### Quantitative real-time PCR and immunohistochemistry

The mRNA and protein expression of the key gene in LUAD were determined using quantitative real-time PCR (qRT-PCR) and immunohistochemistry. The clinical tissue samples of LUAD were obtained from patients who received surgery in Thoracic Oncology Department of Sun Yat-sen University Cancer Center, which was approved by the Institutional Review Committee of Sun Yat-sen University Cancer Center. The detailed procedure was performed according to strict adherence to the manufacturers’ instructions.

For qRT-PCR, 24 paired LUAD and normal tissues were resected and stored in RNAlater immediately. Total RNA was extracted using TRIzol reagent. cDNA was synthesized from total RNA using cDNA reverse transcription kit (Thermo Fisher Scientific). qRT-PCR was performed using the SYBR Green PCR kit (Thermo Fisher Scientific). The housekeeping gene *GAPDH* was used as an endogenous control. Primer information: *FANCD2*: 5′- AAAACGGGAGAGAGTCAGAATCA-3′ (forward) and 5′- ACGCTCACAAGACAAAAGGCA-3′ (reverse); *GAPDH*: 5′- GGAGCGAGATCCCTCCAAAAT-3′ (forward) and 5′- GGCTGTTGTCATACTTCTCATGG-3′ (reverse). The cycle threshold (Ct) of each gene in samples was recorded. Relative quantification was calculated as 2-ΔCt (ΔCt values = target gene mean Ct value - control gene mean Ct value).

Twenty pairs of LUAD immunohistochemistry samples were fixed using 10% formalin and embedded in paraffin. Immunohistochemistry was carried out using the processed 5 μm continuous sections. Samples were dewaxed with decreasing concentrations of 100, 95, 75, and 50% ethanol and washed in deionized water. The sections were heated in a microwave with TE buffer pH 9.0 to retrieve antigens. Endogenous peroxidase was inhibited by incubation in goat serum. Then they were incubated in rabbit anti-FANCD2 (Proteintech, 204006–1-AP, 1:200) overnight at 4 °C. Next, the sections were incubated with horseradish peroxidase-coupled goat anti-rabbit secondary antibody and stained using DAB Detection Kit (Polymer). The following process is cell nucleus staining, dehydration, xylene infusion, and mounting [24]. The immunohistochemical scores were scored by two independent pathologists. The intensity of FANCD2 expression was scored as zero, negative; one point, weak staining; two points, mild staining; three points, strong staining. The positive stained area percentage (PSAP) of FANCD2 expression was scored as 1, 0–25%; 2, 25–50%; 3, 50–75% and 4, 75–100%. FANCD2 IHC score = Intensity score × PSAP score.

### Statistical analysis and R software packages

Significance analysis of microarrays was used to screen the differentially expressed genes between the LUAD and normal lung tissues. Univariate Cox proportional hazards model was used to analyze the association between gene expression level and prognosis. Kaplan-Meier method and Log-rank test were used to evaluate the difference between survival curves. The continuous and categorical variables between the two risk subtypes were compared using the two-sided Wilcoxon rank-sum test and chi-square test, respectively. Benjamini-Hochberg method was used to adjust for multiple hypothesis testing. All *P* values were 2-sided, and *P* < 0.05 was considered statistically significant.

Statistical analyses and result visualization were performed using R software v3.6.3, v4.0.5 and v4.1.2 (“pheatmap v4.0.5”, “limma v3.6.3 [25]”, “survival v3.6.3”, “survminer v3.6.3”, “ggpubr v3.6.3”, “survivalROC v3.6.3”, “car v3.6.3”, “ggridges v3.6.3”, “genefilter v4.1.2”, “ggpubr v3.6.3”, “pRRophetic v4.1.2”, “ggplot2 v3.6.3”, “colorspace v4.0.5”, “stringi v4.0.5”, “clusterProfiler v4.1.2 [26]”, “enrichplot v4.1.2” and “maftools v4.1.2” R package).

## Results

### Characteristics of the LUAD patients from datasets

**Fig. 1:**
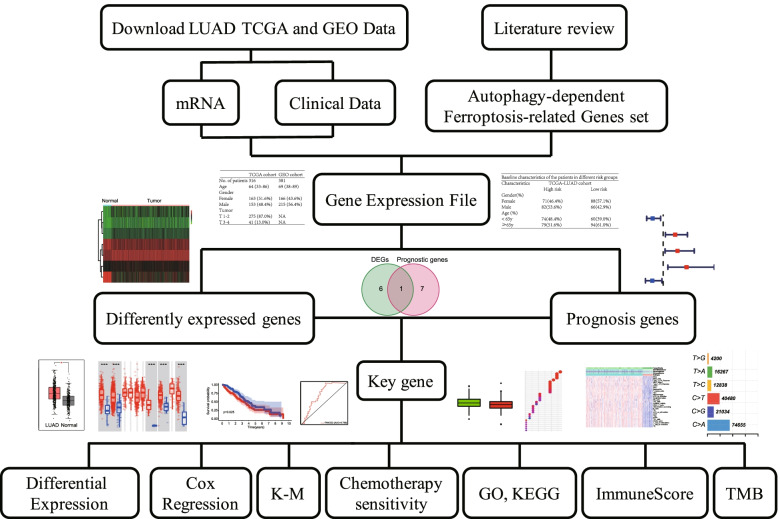
A research framework for the exploration procedure and comprehensive analysis of the autophagy-dependent ferroptosis-related gene. LUAD, Adenocarcinoma of lung; TCGA, The Cancer Genome Atlas; GEO, Gene Expression Omnibus; mRNA, messenger RNA; K-M, Kaplan-Meier; GO, Gene Ontology; KEGG, Kyoto Encyclopedia of Genes and Genomes; TMB, Tumor Mutational Burden

**Fig. 2:**
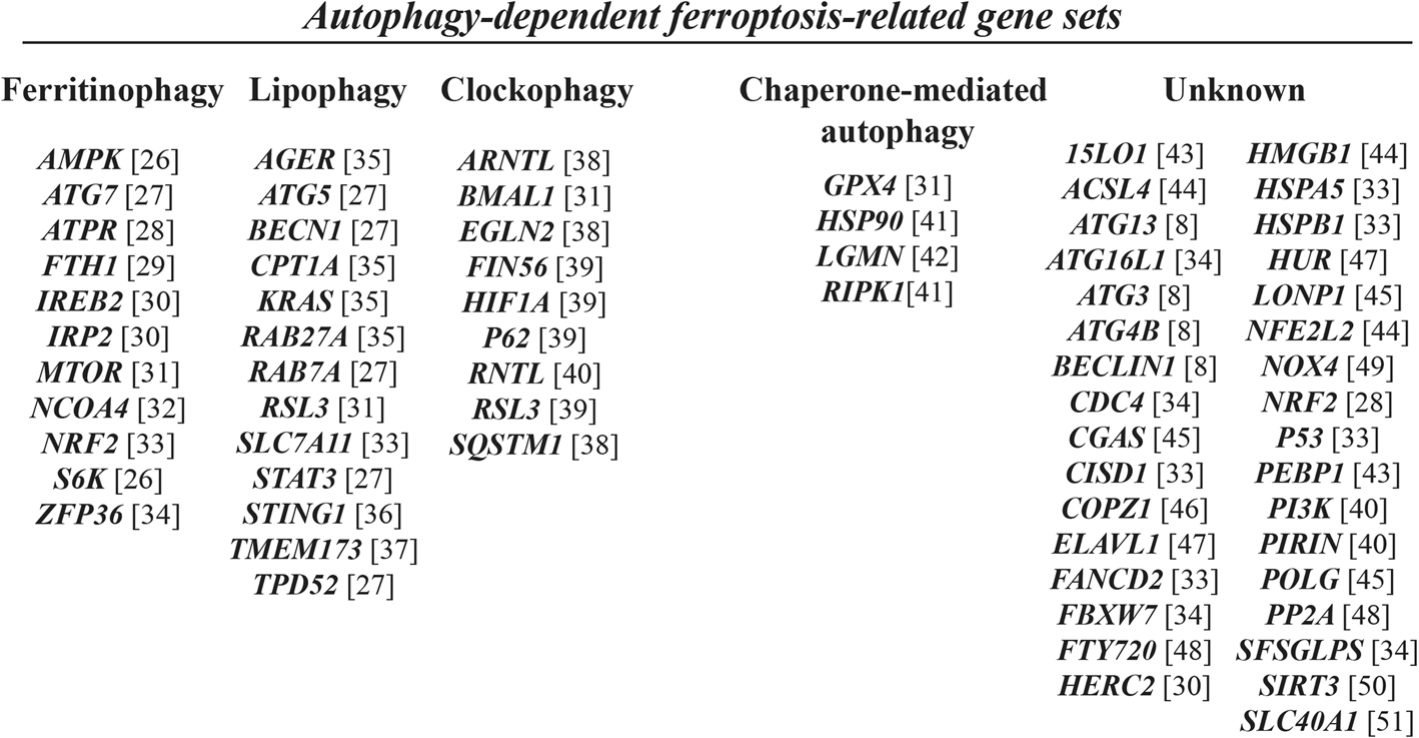
Autophagy-dependent ferroptosis-related gene sets which were identified from literature

The flow chart of this study is shown in Fig. 1. 316 LUAD patients from the TCGA cohort and 381 LUAD patients from the GEO (GSE116959) cohort were finally enrolled. The detailed clinical and tumor characteristics of the LUAD cohorts are summarized in Table 1. A total of 70 autophagy-dependent ferroptosis-related genes were identified from literatures and showed in Fig. 2 [27–31]. These genes were classified into ferritinophagy, lipophagy, clockophagy, chaperone-mediated autophagy, and others according to the autophagy type.

**Table 1:** Clinical and tumor characteristics of the LUAD cohorts

	TCGA cohort	GEO cohort
**No. of patients**	316	381
**Age (median, range)**	64 (33–86)	69 (38–89)
**Gender**		
Female	163 (51.6%)	166 (43.6%)
Male	153 (48.4%)	215 (56.4%)
**Tumor**		
T 1–2	275 (87.0%)	NA
T 3–4	41 (13.0%)	NA
**Node**		
**N** 0–1	267 (84.5%)	NA
**N** 2–3	49 (15.5%)	NA
**Metastasis**		
**M** 0	296 (93.7%)	NA
**M** 1	20 (6.3%)	NA
**TNM stage**		
I	164 (51.9%)	246 (64.6%)
II	76 (24.1%)	65 (17.1%)
III	56 (17.7%)	56 (14.7%)
IV	20 (6.3%)	14 (3.6%)
***TP53***		
Wide type	NA	287 (75.3%)
Mutant type	NA	94 (24.7%)
**Survival status**		
OS years (median)	2.19	2.24

*LUAD* adenocarcinoma of lung, *TCGA* The Cancer Genome Atlas, *GEO* Gene Expression Omnibus, *No* number, *T* tumor, *N* regional lymph node, *M* metastasis, *NA* not available, *OS* overall survival

**Fig. 3:**
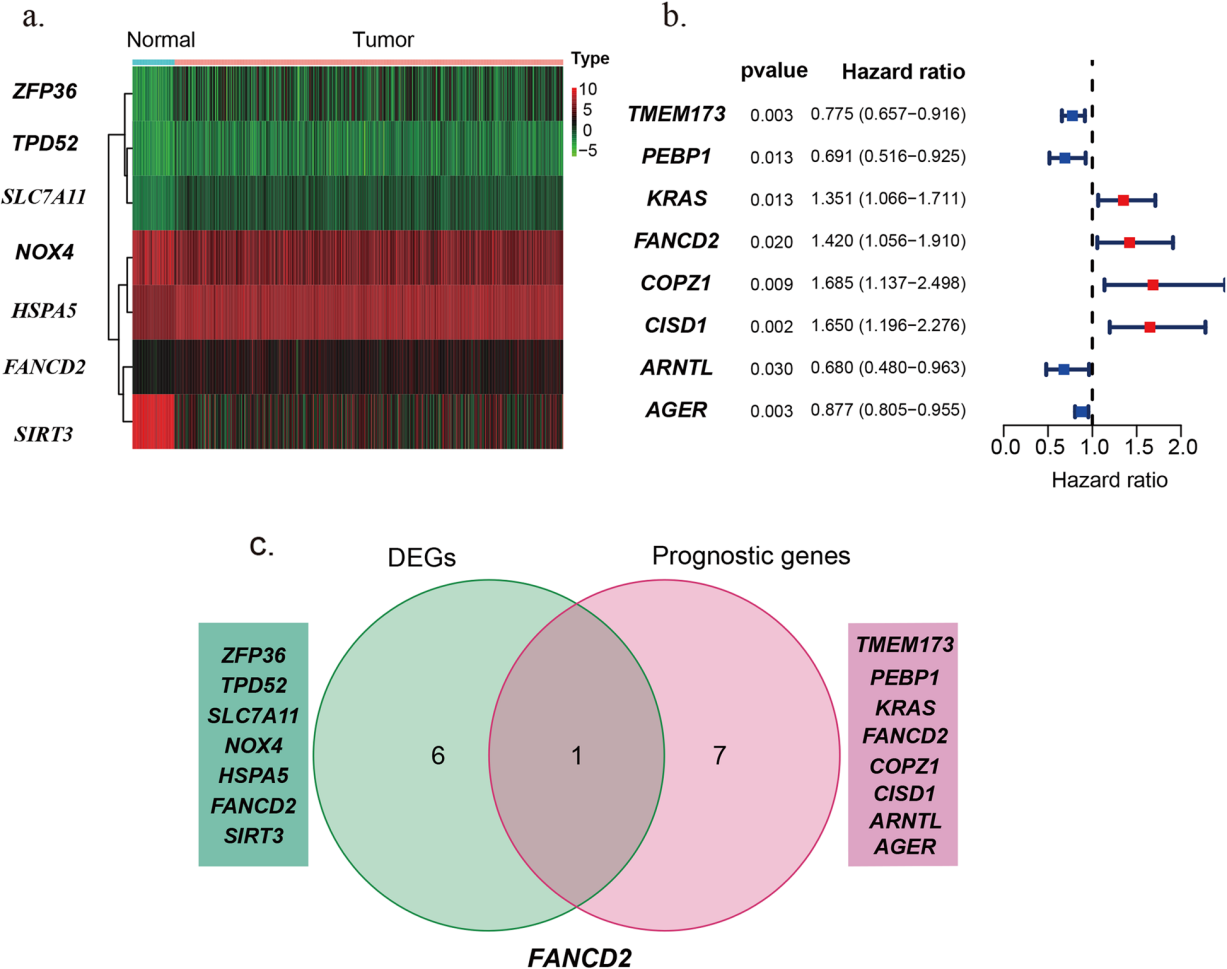
The identification of the key gene from the autophagy-dependent ferroptosis-related genes in the TCGA-LUAD cohort. **a** There are 7 DEGs that were differentially expressed in LUAD and normal lung tissue. **b** There are 8 prognostic genes that were related to the overall survival of LUAD. **c** *FANCD2* is identified as the key gene differentially expressed and prognostic-related to LUAD. DEGs, Differentially Expressed Genes

Three steps were carried out to screen the key gene. First, 7 DEGs in Fig. 3a were selected. Among the 7 DEGs, *CBS*, *CHAC1*, *DPP4*, *FANCD2* and *GCLC* are highly expressed in LUAD tissues (logFC > 1, *P* < 0.05). However, *ALOX15* and *ALOX5* are lowly expressed (logFC < 1, *P* < 0.05). Second, 8 genes with prognostic values for LUAD were selected (Fig. 3b). Among the eight prognosis genes, the high expression level of *KRAS*, *FANCD2*, *COPZ1*, and *CISD1* were related to poor survival of LUAD (Hazard ratio > 1 and P < 0.05). *TMEM173*, *PEBP1*, *ARNTL* and *AGER* were identified as prognosis protective genes (Hazard ratio < 1 and *P* < 0.05). Third, the key gene was obtained as the intersection between DEGs and prognosis-related genes using Venn diagrams (Fig. 3c). As a result, *FANCD2* was identified as the key gene, which worked as a prognosis-related differentially expressed gene in LUAD.

### The mRNA and protein expression levels of *FANCD2* are higher in LUAD than in normal lung samples

**Fig. 4:**
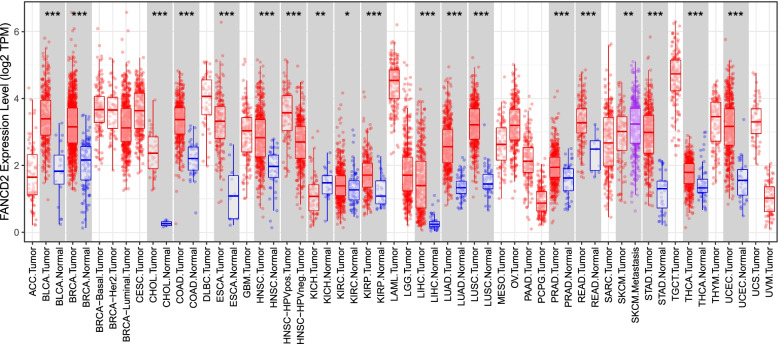
The transcription levels of *FANCD2* in different types of cancers in TIMER database. TIMER: The Tumor IMmune Estimation Resource

**Fig. 5:**
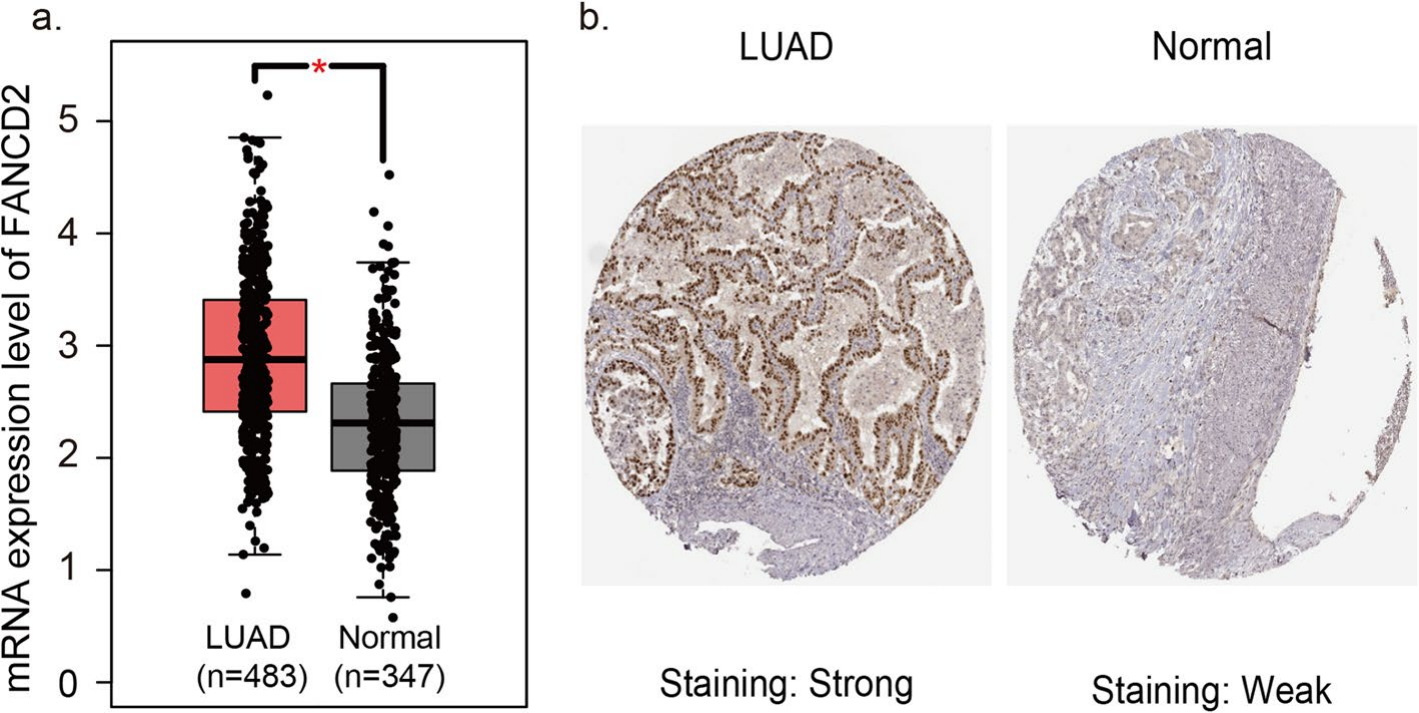
The expression levels of *FANCD2* in LUAD and normal lung tissues in databases. **a** The mRNA expression level of *FANCD2* in GEPIA database. **b** The protein expression level of FANCD2 in HPA database. GEPIA, Gene Expression Profiling Interactive Analysis; HPA, The Human Protein Atlas; N, number

**Fig. 6:**
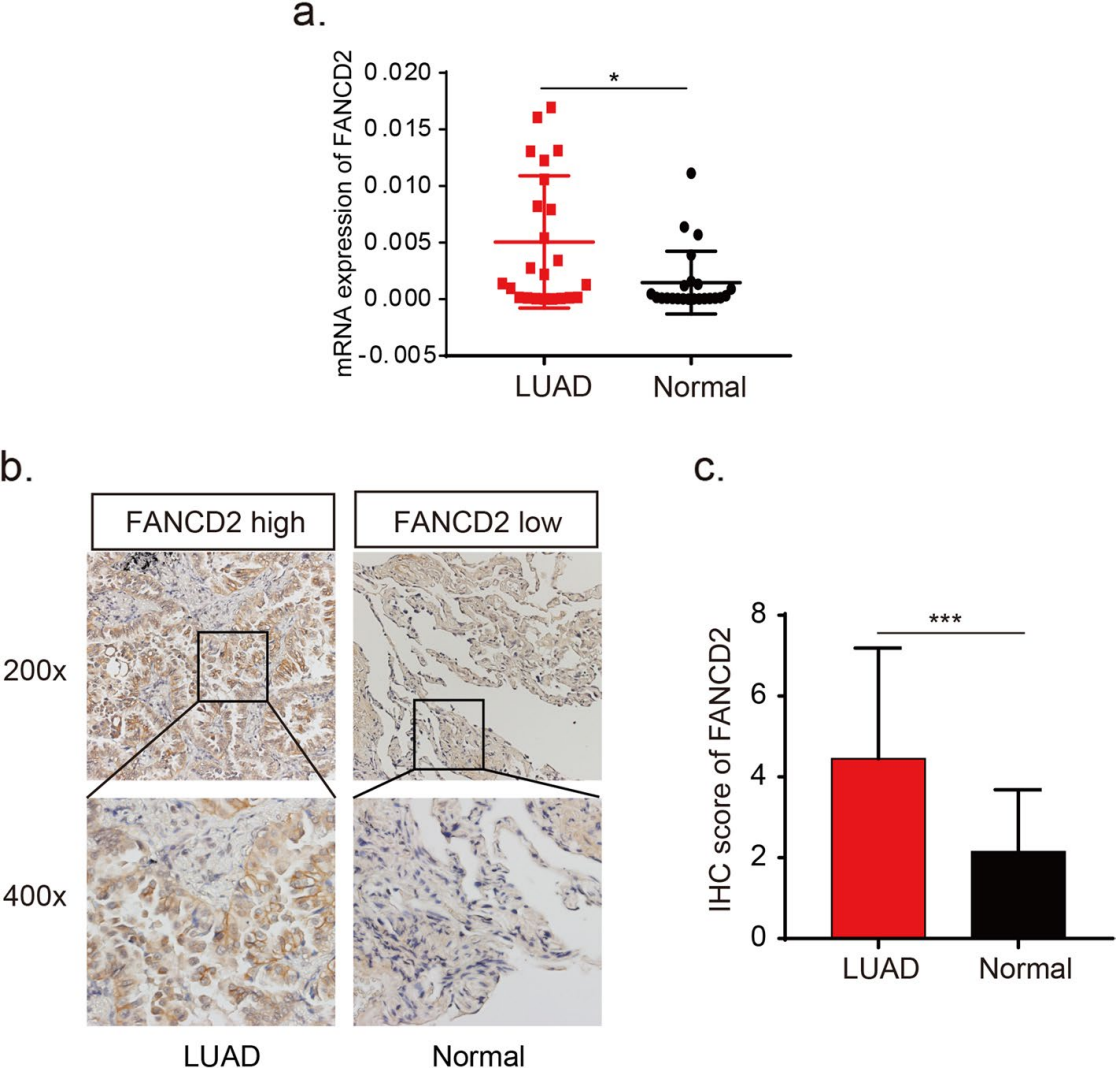
The expression levels of *FANCD2* in LUAD and normal lung tissues in the SYSUCC cohort. **a** The mRNA expression level of *FANCD2* is higher in LUAD than normal lung tissues by qRT-PCR. **b** The typical immunohistochemical images of FANCD2 in LUAD and normal lung tissues. **c** The immunohistochemical score of FANCD2 is higher in LUAD than in normal lung tissues. qRT-PCR, Real-Time Quantitative RT-PCR; IHC: immunohistochemistry

The *FANCD2* expression in different tumors was evaluated using TCGA RNA-sequencing data (Fig. 4). *FANCD2* expression was significantly higher in various tumors than adjacent normal tissues, and the consistent findings were shown in LUAD (Fig. 5a). qPCR results provided the biological evidence by using 24 pairs of LUAD and sample lung tissues (Fig. 6a). After examining the mRNA expression level of *FANCD2* in LUAD, the protein expression level was further explored by immunohistochemistry. The grading standards of the IHC score are shown in Supplementary Fig. 1. There is a higher IHC score of FANCD2 in LUAD tissues than normal lung tissues (Fig. 6b, c), which is in line with HPA database (Fig. 5b). In summary, the present results indicated that both transcriptional and translational expression levels of *FANCD2* were overexpressed in patients with LUAD, which may be involved in the pathogenesis of LUAD.

### Prognostic risk model and predictability evaluation

**Fig. 7:**
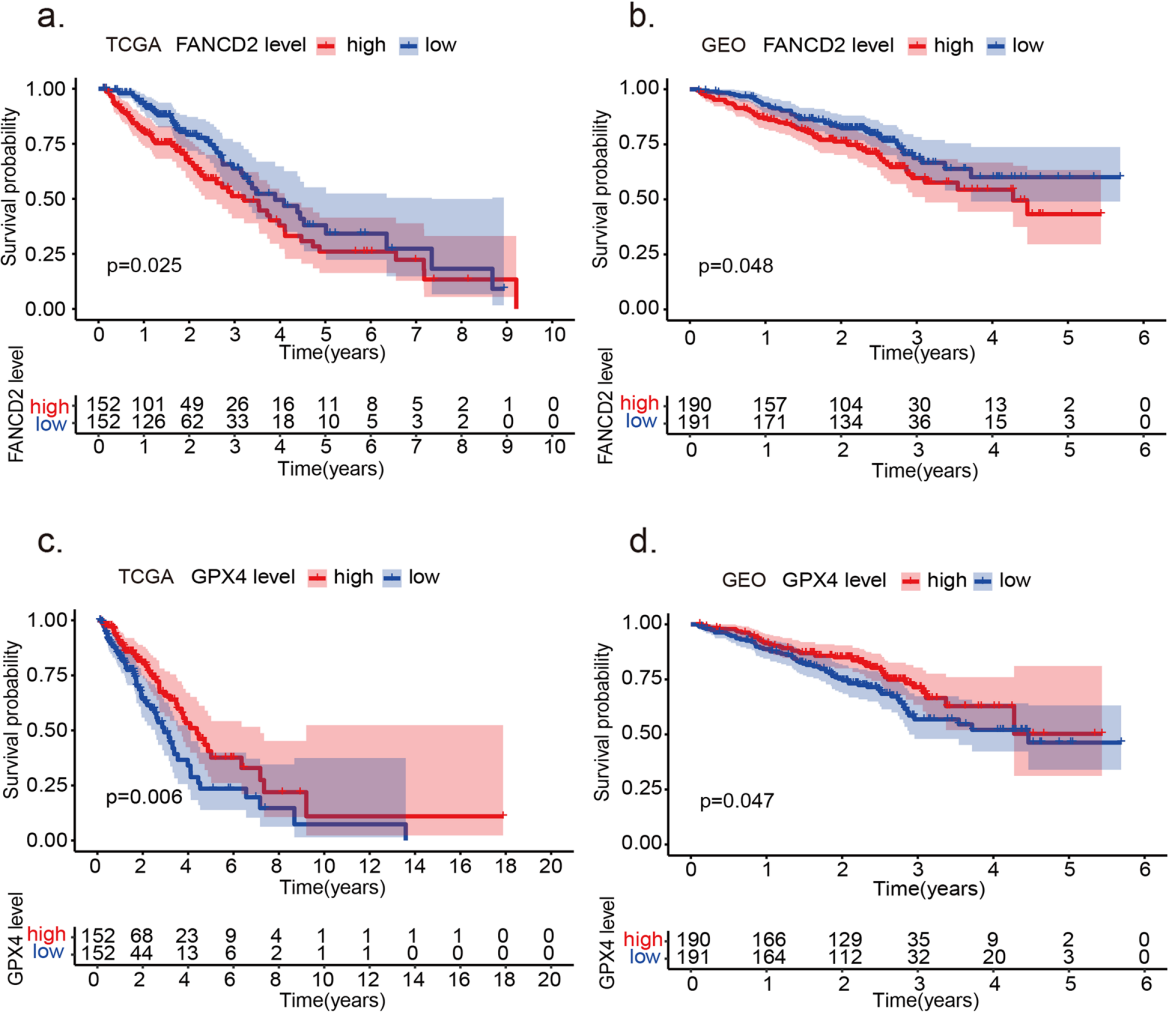
Kaplan-Meier survival analysis of *FANCD2* and *GPX4* in TCGA-LUAD and GEO-LUAD cohort. **a** The high expression of *FANCD2* is related to poor survival in the TCGA-LUAD cohort. **b** The high expression of *FANCD2* is associated with poor survival in the GEO-LUAD cohort. **c** The high expression of *GPX4* is associated with good survival in the TCGA-LUAD cohort. **d** The high expression of *GPX4* is related to good survival in the GEO-LUAD cohort

The LUAD patients were stratified into high and low-risk groups by the median *FANCD2* expression level. The low-risk group is well matched by propensity score matching to the high-risk group both in TCGA and GEO cohort (Supplementary Table 3 and Supplementary Table 4). Table 2 shows the association of *FANCD2* expression and the clinical features. A high expression of *FANCD2* achieved a significant correlation with a high TNM stage (*P* < 0.05). In the TCGA cohort, the *FANCD2* expression was defined as an independent prognostic factor after the univariate and multivariate Cox regression analyses (Fig. 7a). The patients in high-risk groups have a poor survival than the low-risk group in the TCGA cohort.

**Table 2:** Baseline characteristics of the patients in different risk groups

Characteristics	TCGA-LUAD cohort			GEO-LUAD cohort		
	High risk	Low risk	***P*** value	High risk	Low risk	***P*** value
**Gender(%)**			0.09			0.25
Female	71 (46.4%)	88 (57.1%)		112 (58.9%)	103 (53.9%)	
Male	82 (53.6%)	66 (42.9%)		78 (41.1%)	88 (46.1%)	
**Age (%)**			0.17			0.99
< 65y	74 (48.4%)	60 (39.0%)		52 (27.4%)	52 (27.2%)	
≥ 65y	79 (51.6%)	94 (61.0%)		138 (72.6%)	139 (72.8)	
**TNM stage**			**0.03**			**0.01**
I-II	112 (73.2%)	120 (77.9%)		152 (80.0%)	159 (83.2%)	
III-IV	41 (26.8)	34 (22.1%)		38 (20.0%)	32 (16.7%)	
***TP53***			NA			**< 0.001**
Wide type	–	–		132 (69.5%)	155 (81.1%)	
Mutant type	–	–		58 (30.5%)	36 (18.8%)	

*LUAD* adenocarcinoma of lung, *TCGA* The Cancer Genome Atlas, *GEO* Gene Expression Omnibus, *y* years, *NA* not available

**Fig. 8:**
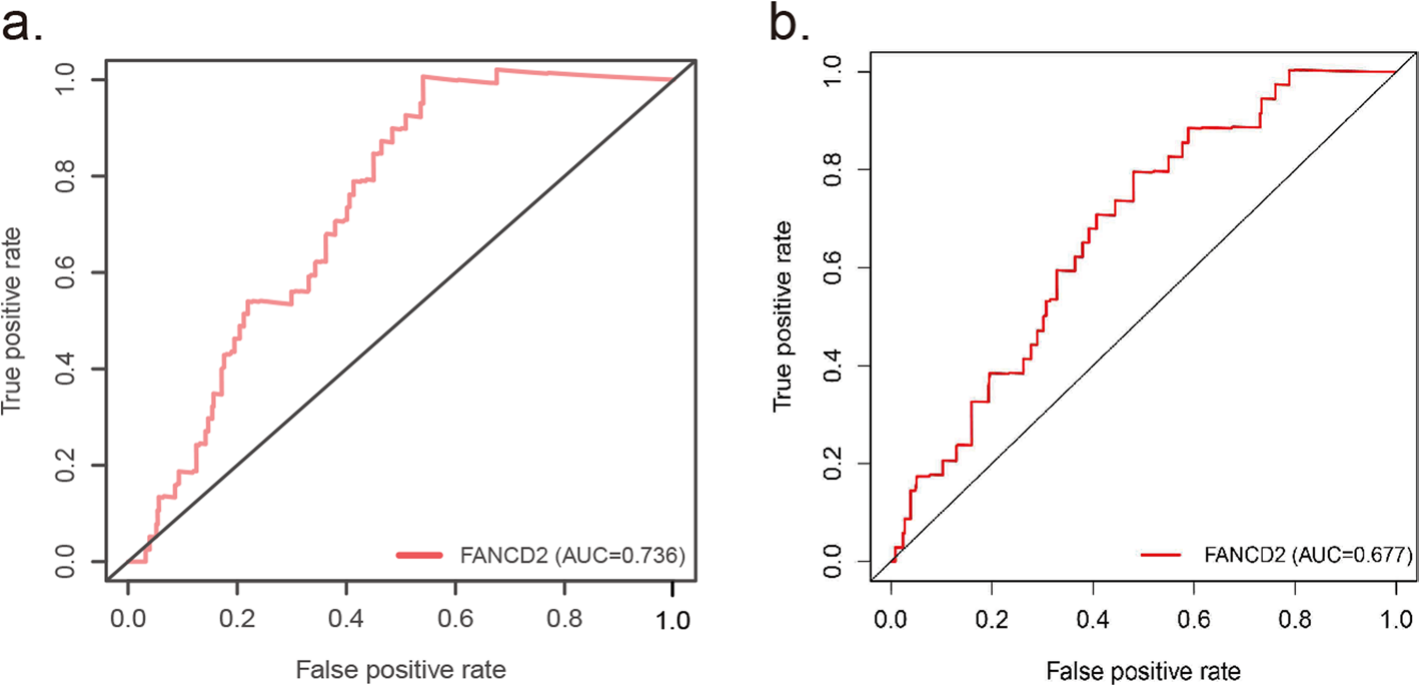
ROC curve of the *FANCD2* risk score in TCGA-LUAD and GEO-LUAD cohort. The risk score is shown by the receiver operating characteristic curve for predicting survival of the TCGA cohort (**a**) and the GEO cohort (**b**). ROC curve, receiver operating characteristic curve

**Fig. 9:**
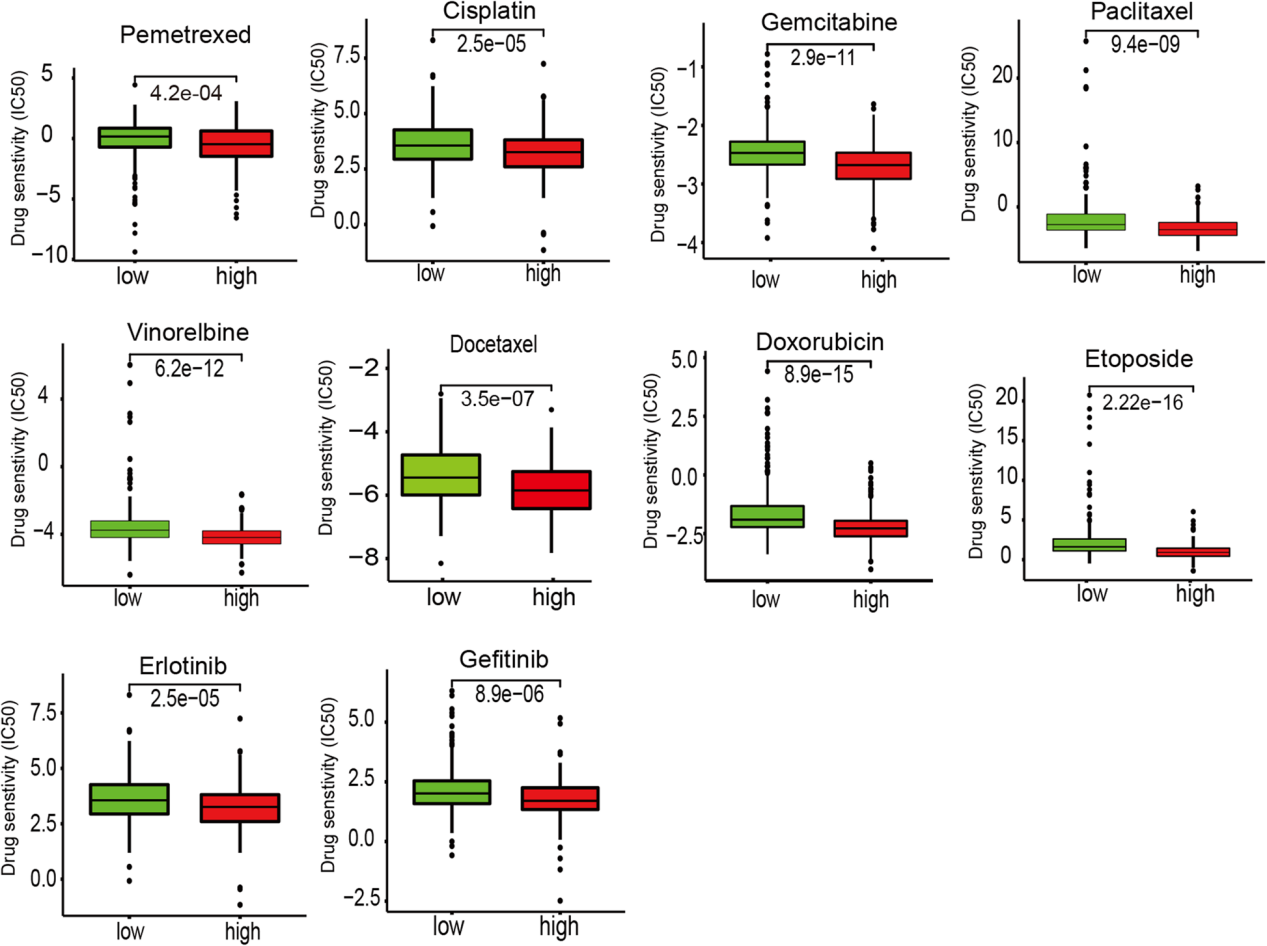
Chemotherapeutic response prediction of 29 chemotherapies in the high- and low-*FANCD2* groups in TCGA-LUAD cohort

Similarly, relevant data from a GEO cohort (GSE35570) was used to validate the prognostic value of *FANCD2* expression in LUAD (Fig. 7b). And the high expression of ferroptosis regulator *GPX4* is related to a better prognosis in LUAD patients both in TCGA (*P* = 0.006) and GEO database (*P* = 0.006) (Fig. 7c, d). Besides the poor survival and a high TNM stage, the increased expression of *FANCD2* was also related to a high frequency of TP53 mutation (*P* < 0.001, Table 2). The sensitivity and specificity of the *FANCD2* model were calculated by the area under ROC (TCGA cohort: AUC = 0.736, GEO cohort: AUC = 0.677), suggesting that the *FANCD2* signature was adequate for predicting survival of LUAD (Fig. 8). We investigated the response to chemotherapy in high- and low-risk patients with LUAD. We found that 29 chemotherapeutic drugs displayed significant differences in estimated IC50 between high and low-risk patients and that high-risk patients showed increased sensitivity to all 29 chemotherapies (Fig. 9).

### Functional annotation of DEGs in different risk groups

**Fig. 10:**
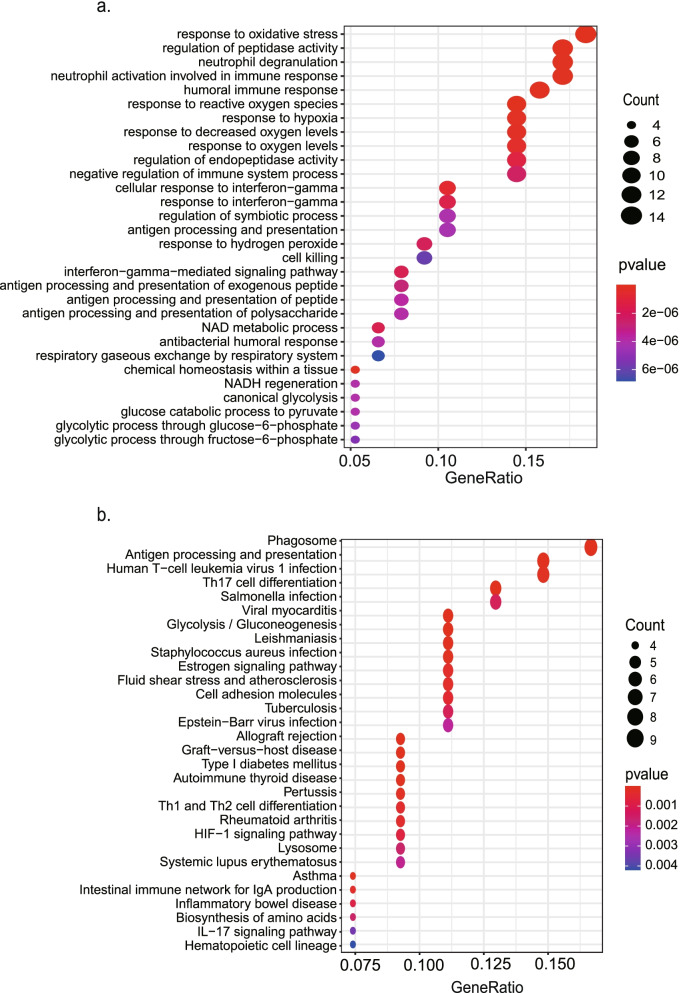
Representative results of GO (**a**) and KEGG analyses (**b**) of the high- and low-*FANCD2* groups in the TCGA-LUAD cohort. GO, Gene Ontology; KEGG, Kyoto Encyclopedia of Genes and Genomes

GO and KEGG pathway enrichment analyses were used to evaluate the possible functions and pathways of the DEGs. There is a total of 80 DEGs between the high and low expression level of *FANCD2* in the TCGA-LUAD cohort, which are shown in Supplementary Table 7. The top five GO terms were “response to reactive oxygen species”, “regulation of peptidase activity”, “neutrophil degranulation”, “neutrophil activation involved in immune response”, and “neutrophil activation” (Fig. 10 a). The top five pathways were “phagosome”, “antigen processing and presentation”, “human T-cell leukemia virus”, “Th17 cell differentiation”, and “salmonella infection” by KEGG enrichment (Fig. 10 b).

The results of GO analysis showed that these DEGs might be involved in response to reactive oxygen species and immune processes. The data were consistent with our results that *FANCD2* is correlated with ferroptosis and immune responses. Pathway enrichment analysis revealed that DEGs might be enriched in pathways related to phagosome and antigen processing and presentation, indicating that these genes function in autophagy and immune system.

**Fig. 11:**
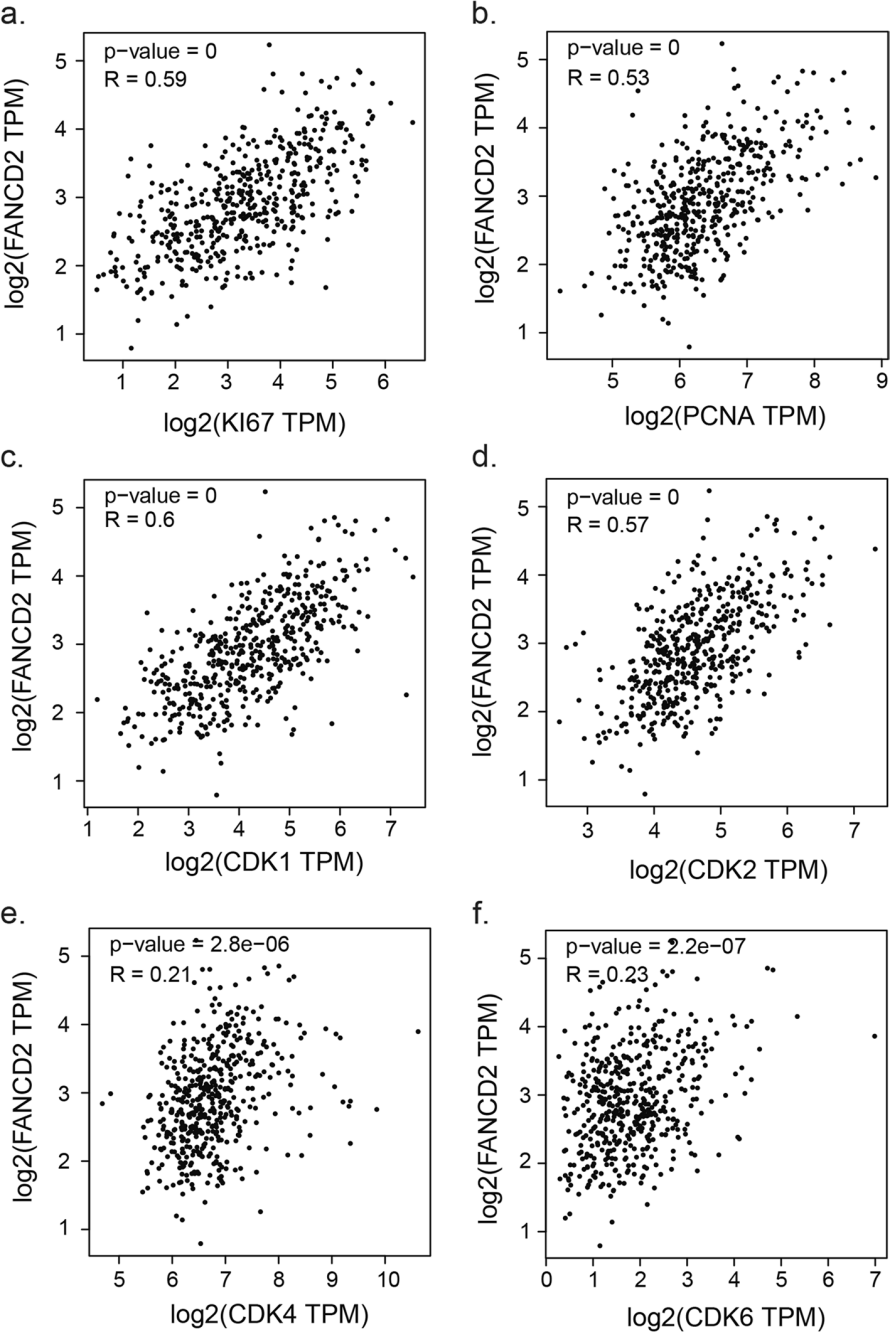
The correlation analysis of *FANCD2* with tumor proliferation and cell cycle markers in GEPIA database

In the correlation analysis we can see that *FANCD2* is associated with tumor proliferation markers, including *KI67* (*R* = 0.59, *P* < 0.001), *PCNA* (*R* = 0.53, *P* < 0.001), and cell cycle markers, incluidng *CDK1* (*R* = 0.60, *P* < 0.001), *CDK2* (*R* = 0.57, *P* < 0.001), *CDK4* (*R* = 0.21, *P* < 0.001), *CDK6* (*R* = 0.23, *P* < 0.001) (Fig. 11).

### Association between *FANCD2* and immune-related scores

**Fig. 12:**
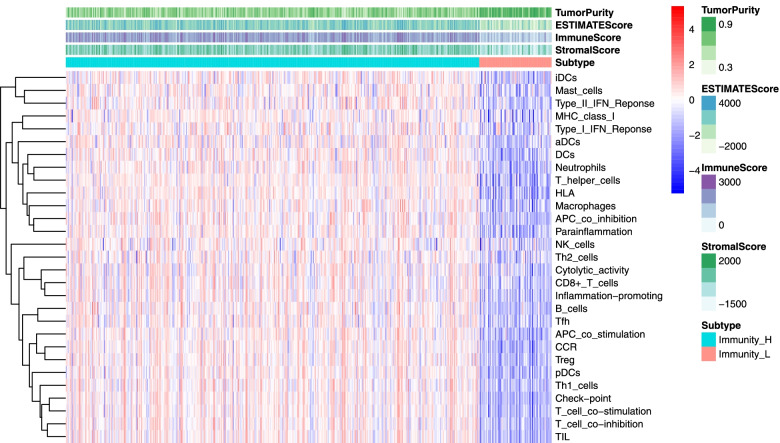
The enrichment levels of immune-related process and immune score in TCGA-LUAD cohort

**Fig. 13:**
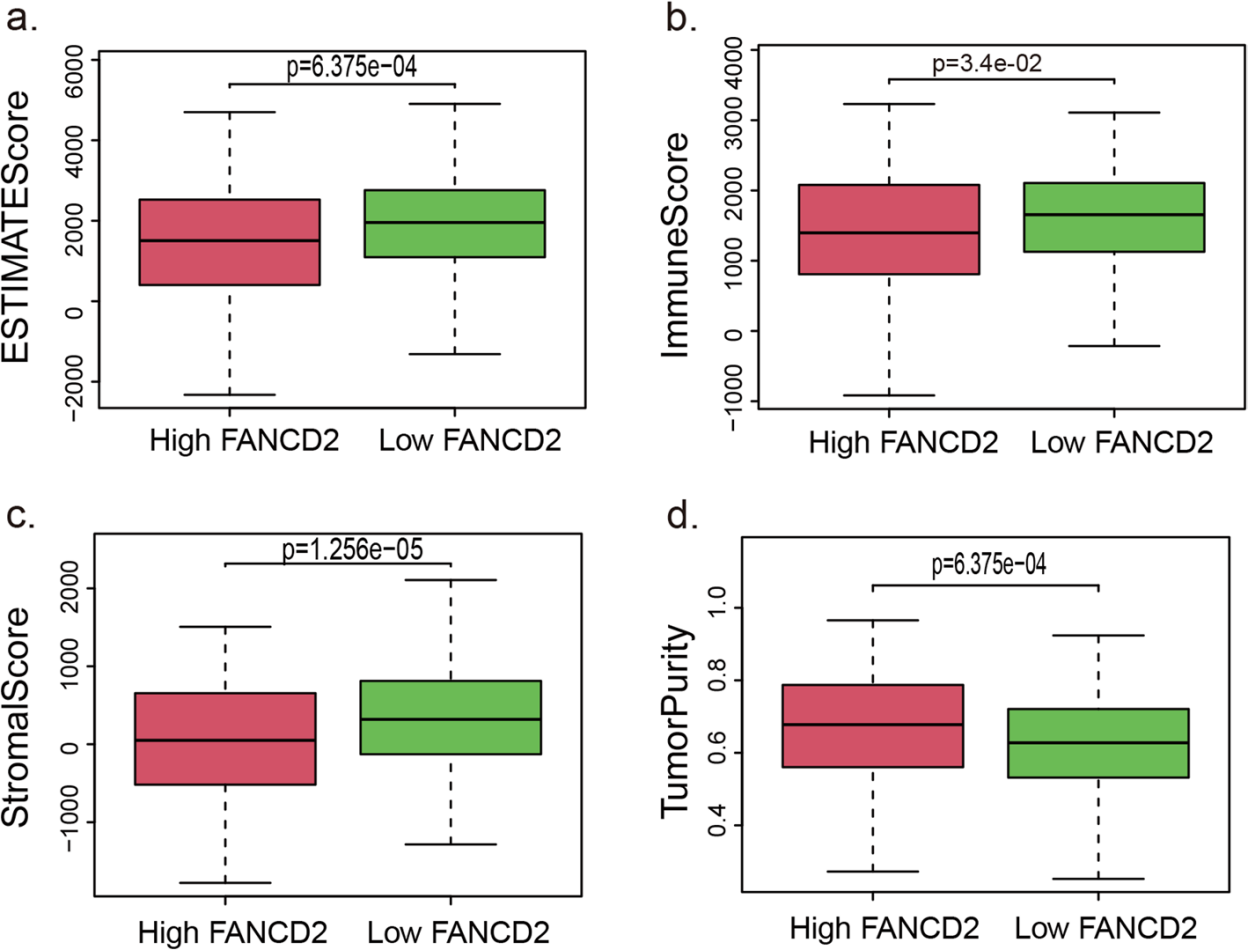
The correlation between *FANCD2* and ESTIMATE Score (**a**), Immune Score (**b**), Stromal Score (**c**), Tumor Purity (**d**) which calculated by ssGSEA. ssGSEA: Single Sample Gene Set Enrichment Analysis

**Fig. 14:**
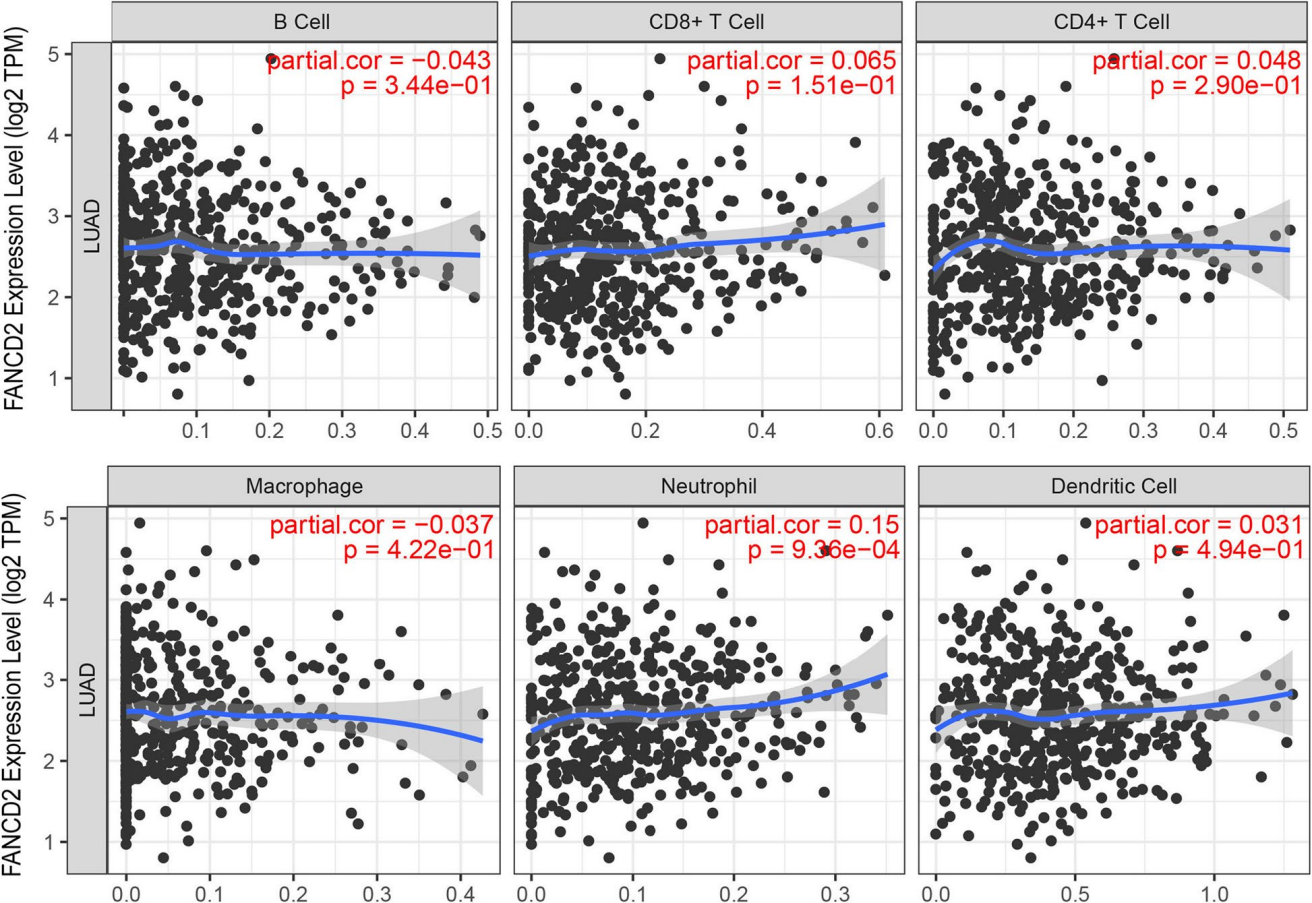
The correlation between the *FANCD2* expression level and six immune cells infiltration level in TIMER database

The potential immune mechanisms of LUAD were further explored through the scoring of tumor immune components (TumorPurity, ESTIMATEScore, ImmuneScore, StromalScore) and immune infiltrating cells counted according to immunity-enriched groups (Fig. 12). Each patient in the LUAD cohort was scored by the above indicators. The immune cell infiltration levels changed along with the *FANCD2* gene copy numbers (Fig. 13). The LUAD patients with a high expression level of *FANCD2* had a low ESTIMATEScore (Fig. 13a), ImmuneScore (Fig. 13b), and StromalScore (Fig. 13c), but high TumorPurity (Fig. 13d) was found in the high expression of *FANCD2*. Neutrophil cell infiltration levels seemed to associate with altered *FANCD2* gene copy numbers in LUAD positively (cor = 0.15, *P* < 0.001, Fig. 14), which is consistent with the GO results of neutrophil degranulation, neutrophil activation involved in immune response, and neutrophil activation in Fig. 10a.

### The landscape of mutation profiles in LUAD cohort

**Fig. 15:**
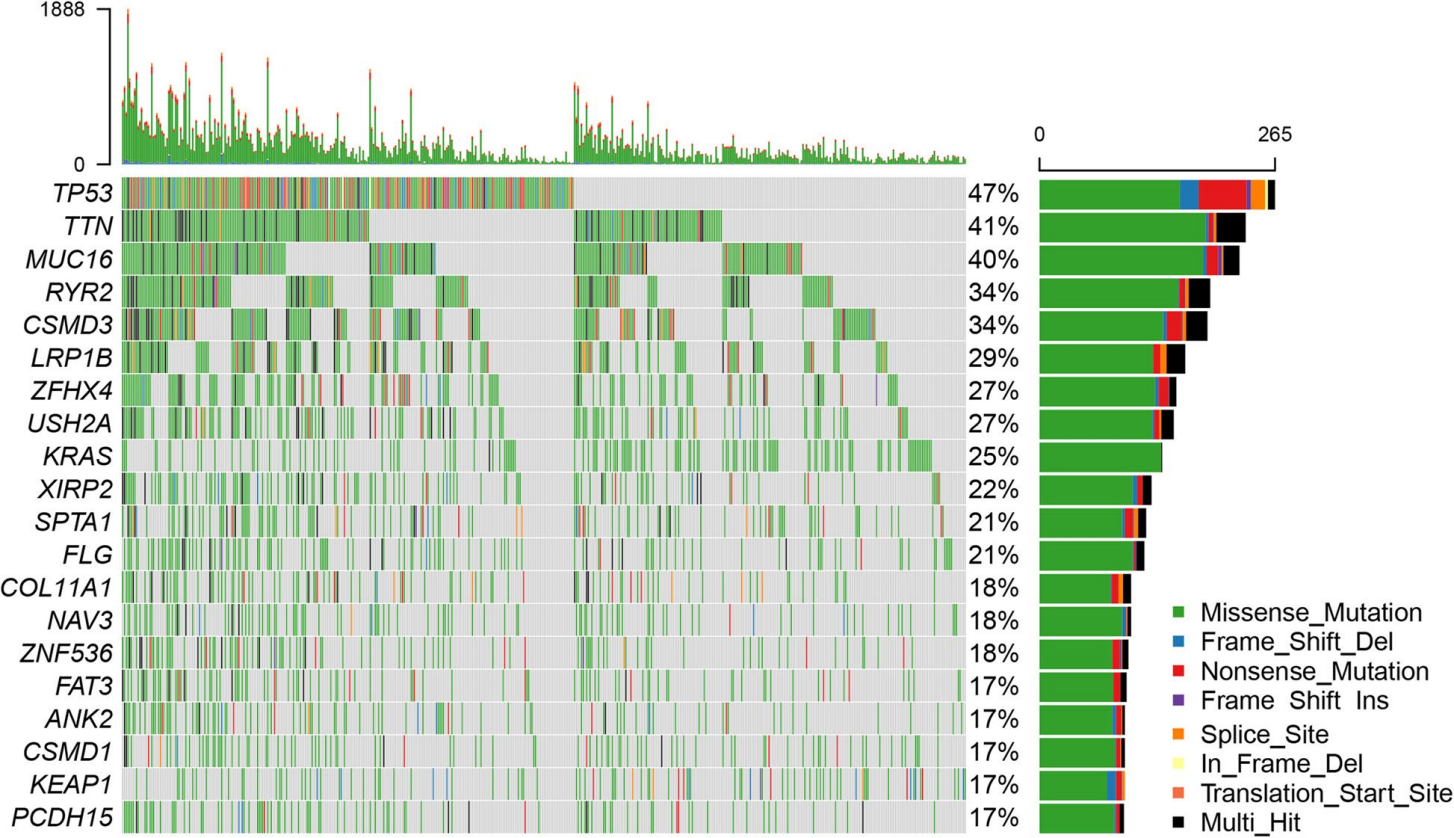
Oncoplot of the most frequently mutated genes with driver mutations in TCGA-LUAD cohort

**Fig. 16:**
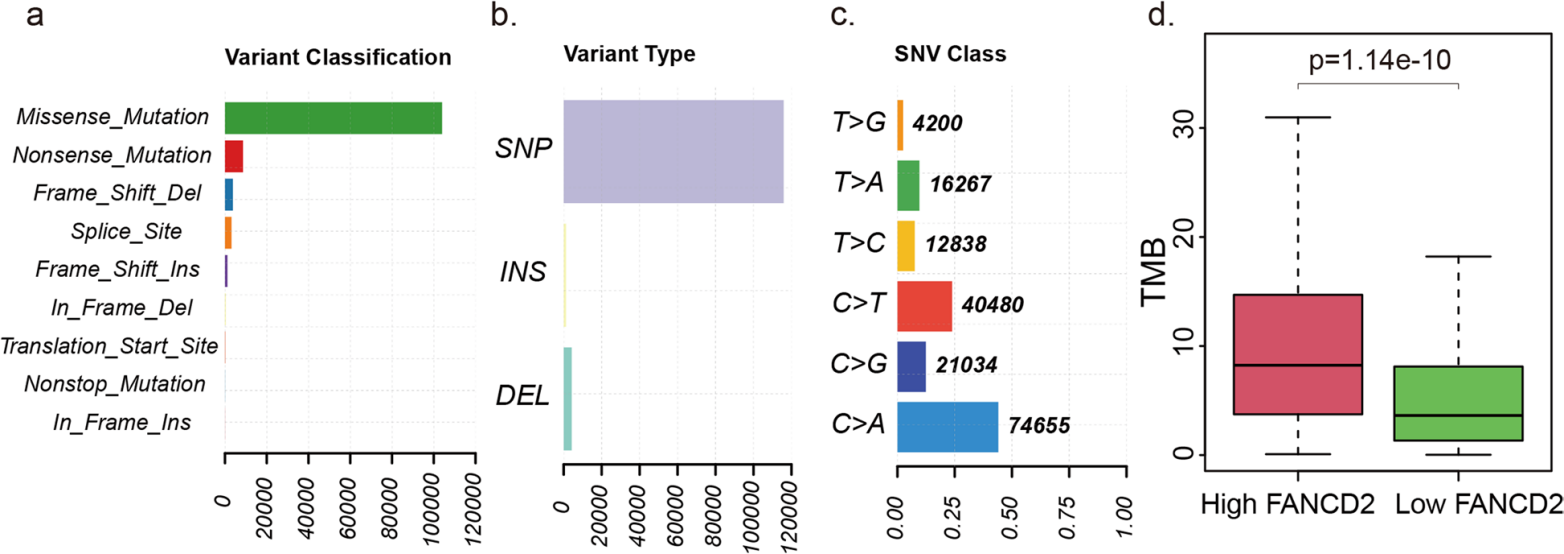
The Variant Classification (**a**), the Variant Type (**b**), the Single Nucleotide Variant Class (**c**) and the correlation of the *FANCD2* expression levels with TMB (**d**) in the TCGA-LUAD cohort. SNP, single nucleotide polymorphism; INS, insertion; DEL, deletion; SNV, single nucleotide variants; TMB, tumor mutational burden

The most frequent driver mutations of the LUAD cohort were displayed in oncoplot, where various colors with annotations represented the different mutation types (Fig. 15). Then, the top 5 mutated genes in LUAD with ranked percentages were exhibited, including *P53* (47%), *TTN* (41%), *MUC16* (40%), *RYR2* (34%), and *CSMD3* (34%). These mutations were further classified according to different mutation categories. Findings indicated that missense mutation accounted for the most fraction (Fig. 16a), and single nucleotide polymorphism (SNP) occurred more frequently than insertion or deletion (Fig. 16b), and C > A was the most common single nucleotide variants (SNV) in LUAD (Fig. 16c). The LUAD patients with a high expression level of *FANCD2* showed a higher TMB (*P* < 0.001, Fig. 16d), which suggested that *FANCD2* could work as a TMB marker and play a role in prediction of response to immunotherapy.

## Discussion

LUAD is a common malignancy with high morbidity and mortality [1]. The development of LUAD often involves genetic abnormalities and immune dysfunction [32]. Iron metabolism could influence malignant biological behaviors and impact the tumor microenvironment [33]. The increase of labile iron in cancer cells can facilitate DNA replication [34] and induce the occurrence of ferroptosis to participate in and accelerate tumor progression [4].

Ferroptosis is a programmed cell death in which multiple signaling molecules interact in the tumor microenvironment and synergistically regulate tumor progression [35]. Ferroptosis has a dual role in tumor promotion and suppression [36]. On the one hand, the induced tumor cell ferroptosis inhibit tumor metastases, is involved in drug resistance, and influences cancer immunotherapeutic efficacy [37, 38] . On the other hand, ferroptotic damage could contribute to inflammation-related immunosuppression within the tumor microenvironment and promote tumors’ growth [36, 39].

The role of ferroptosis in LUAD has not been elaborated. Our research provides a new perspective for the development of LUAD. Ferroptosis was once considered a novel cell death process distinct from apoptosis, necrosis, and autophagy [4]. However, studies from autophagy-deficient cells suggested that ferroptosis was a type of autophagy-dependent cell death in some conditions [8]. Autophagy, including ferritinophagy [40, 41], lipophagy [28], clockophagy [42, 43], and chaperone-mediated autophagy [42], could promote ferroptosis through lipid peroxidation. As we know, *GPX4* is an essential regulator of ferroptotic cancer cell death [44]. *GPX4* is closely related to the tumor stage and promotes acquired chemoresistance by suppressing ferroptosis [45]. GPX4 inhibitor could augment the anticancer effect of platinum drugs in lung cancer brain metastasis. And GPX4 inhibition synergizes with radiation to induce ferroptosis in LUAD by enhancing cytoplasmic lipid peroxidation [46]. Our study shows that the high expression of *GPX4* is related to a better prognosis in LUAD patients. And the functional analysis of *FANCD2* is mainly enriched in response to oxidative stress and ROS, which indicated the role of ferroptosis in LUAD.

FANCD2 (FA complementation group D2) contributes heterogeneously to Fanconi anemia (FA), a genetic disorder characterized by birth defects, progressive bone marrow failure, and cancer-prone phenotype [47]. The patients with aberrant expression of FANCD2 possess abnormality in chromosomal breakage and hypersensitivity to DNA crosslinking agents [48]. As a DNA damage response regulator, FANCD2 also regulates ferroptosis sensitivity by inhibiting iron accumulation and lipid peroxidation in an autophagy-independent manner [49, 50]. FANCD2 has an intricate relationship with tumors. The heterozygous and somatic mutations of *FANCD2* were reported in various malignancies, including pancreatic cancers and squamous cell carcinomas [51, 52]. The overexpression of *FANCD2* was involved in metastasis-prone melanomas [53] and colorectal cancer [54]. In our study, *FANCD2* was identified as an autophagy-dependent ferroptosis-related key gene in the LUAD occurrence after comprehensive analysis. However, the exact mechanism of how *FANCD2* influences LUAD outcome is complex, which probably includes more than its role in ferroptosis, DNA damage and cell cycle.

Immune cells could regulate tumor ferroptosis during cancer immunotherapy [3]. Besides, ferroptosis also could regulate immunity activity within the tumor microenvironment [39]. The potential connection between the behavior of immune cells in the tumor microenvironment and ferroptosis needs to be further studied. In our study, the high expression of *FANCD2* group achieved a high fraction of neutrophil, which revealed that the ferroptosis-related gene *FANCD2* might be closely associated with neutrophil-mediated tumor immunity.

Following the above findings, the antigen processing and presentation contents were enriched by KEGG analyses. In adaptive immunity, neutrophils play a significant role in internalizing antigen and regulating antigen-specific responses [55]. When ferroptosis occurred, the dead cells released the immunogenic signals, such as lipid mediators. Subsequently, the antigen-presenting cells, including neutrophils, were attracted to the site of ferroptotical cells [39]. A multitude of recruited neutrophils further activated the immune system to resist the invasion of pathogenic factors. Abnormal and uncontrolled ferroptosis may be implicated with invalid immunity [39]. Our research indicated that high expression of *FANCD2* induced aberrant ferroptosis and further contributed to the abnormality of anti-tumor immunity in patients with LUAD.

TMB level has demonstrated utility in selecting patients for response to immunotherapy and has proven to be an essential biomarker for patient selection. Patients in high TMB benefit more from immunotherapy, which provided a new avenue to make LUAD treatment more precise [56]. In our study, the LUAD patients with a high expression level of *FANCD2* achieved a high TMB, indicating that these patients may gain more benefit from immunotherapy than those with a low *FANCD2* expression level. Among the mutation genes, tumor suppressor gene inactivation, such as *P53*, is very common in LUAD [57]. P53 activation has been explored to be essential in some other activities to suppress tumor progression [58, 59], whereas the anti-P53 activity traditionally drives cell senescence, cell cycle arrest, and apoptosis [60].

Additionally, *P53* was correlated with ferroptosis, and it could inhibit cysteine uptake and sensitize cells to ferroptosis. Studies revealed that the sensitivity of ROS-induced ferroptosis was markedly increased in P53-activated cells [61]. Our study found that *P53* is the most frequently mutated gene and positively correlated with a higher *FANCD2* expression level, which indicated that the *P53* mutation might activate the FANCD2-mediated ferroptosis and increase the response of immunotherapy in LUAD.

However, there are several limitations of our study. The role of ferroptosis in LUAD outcome and the role of *FANCD2* in LUAD ferroptosis have not been fully clarified. The function of FANCD2 may include tumor ferroptosis and DNA damage, and cell cycle, which influence the outcome of LUAD together.

## Conclusions

Our study identified a novel autophagy-dependent ferroptosis-related gene, *FANCD2*, which was proved to be independently associated with OS in LUAD and may serve as a prognostic factor for LUAD. The *FANCD2* expression level was negatively correlated with immune infiltrating levels but positively correlated with a somatic mutation in LUAD, which indicated that FANCD2 might act as a potential inhibitor to interfere with immune cells and revealed the possible relationship and interaction between ferroptosis and immunity in LUAD pathogenesis. More mechanistic studies are needed to verify the role and function of FANCD2-mediated ferroptosis in the LUAD in the future.

## Supplementary Information

**Additional file 1.**

**Additional file 2.**

**Additional file 3.**

**Additional file 4.**

**Additional file 5.**

**Additional file 6.**

**Additional file 7.**

**Additional file 8.**

**Additional file 9.**

**Additional file 10.**

**Additional file 11.**

**Additional file 12.**

## Data Availability

The link of TCGA (https://portal.gdc.cancer.gov/repository) and GEO (https://www.ncbi.nlm.nih.gov/geo/query/acc.cgi?acc=GSE116959) data used in the manuscript was provided.

## Abbreviations

LUAD: Lung adenocarcinoma
TMB: Tumor mutation burden
TCGA: The Cancer Genome Atlas
GEO: Gene Expression Omnibus
DEGs: Differentially expressed genes
OS: Overall survival
K-M: Kaplan-Meier
ROC: Receiver operating characteristic
GO: Gene ontology
KEGG: Kyoto encyclopedia of genes and genomes
TIMER: Tumor Immune Estimation Resource
HGNC: HUGO Gene Nomenclature Committee
FDR: False discovery rate
GEPIA: Gene Expression Profiling Interactive Analysis
HPA: The Human Protein Atlas database
IC50: The half maximal inhibitory concentration
ssGSEA: Single Sample Gene Set Enrichment Analysis
SNV: Single-nucleotide variant
qRT-PCR: Real-time quantitative RT-PCR
Ct: Cycle threshold
mRNA: Messenger RNA
FANCD2: FA Complementation Group D2
SNP: Single nucleotide polymorphism
INS: Insertion
DEL: Deletion
