# Supplementary material for: Comprehensive analysis of the autophagy-dependent ferroptosis-related gene FANCD2 in lung adenocarcinoma
Source: BMC Cancer. 2022 Mar 2;22:225. doi: 10.1186/s12885-022-09314-9 (PMC8889748; doi:10.1186/s12885-022-09314-9)
Supplement: Supplementary file 1 — Additional file 1. [file 12885_2022_9314_MOESM1_ESM.pdf]

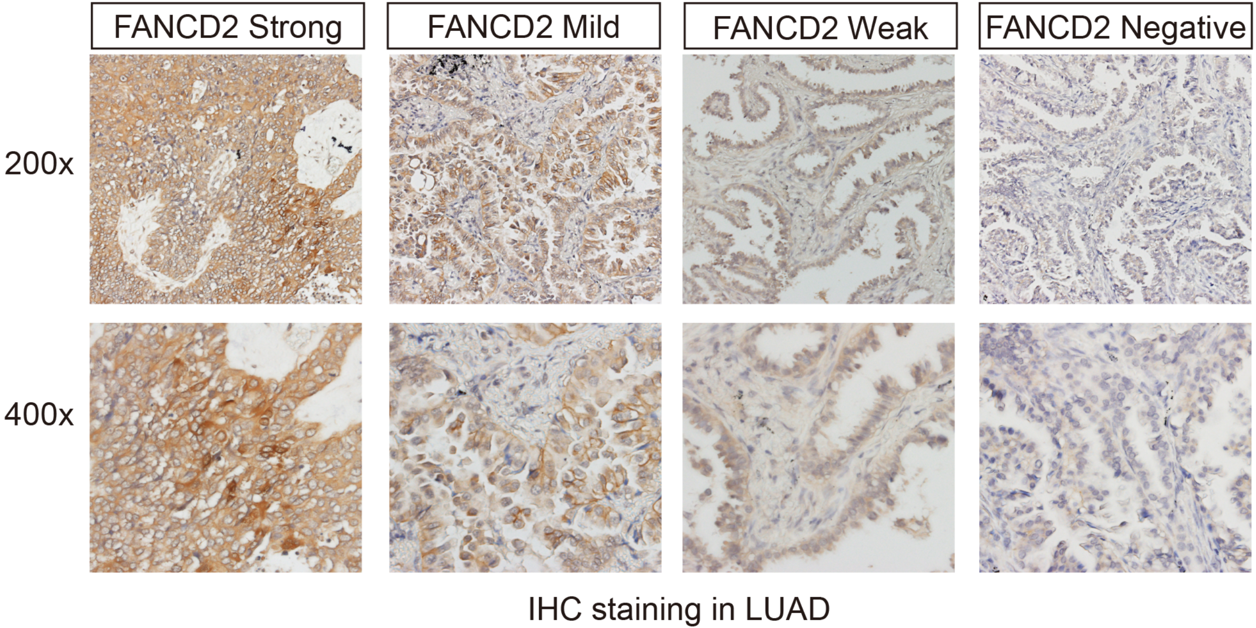

Supplementary Figure 1. The immunohistochemistry score standards of FANCD2 in LUAD. 3, strong staining; 2, mild staining; 1, weak staining and 0, negative staining.
