# Supplementary material for: Comprehensive analysis of the autophagy-dependent ferroptosis-related gene FANCD2 in lung adenocarcinoma
Source: BMC Cancer. 2022 Mar 2;22:225. doi: 10.1186/s12885-022-09314-9 (PMC8889748; doi:10.1186/s12885-022-09314-9)
Supplement: Supplementary file 2 — Additional file 2. [file 12885_2022_9314_MOESM2_ESM.pdf]

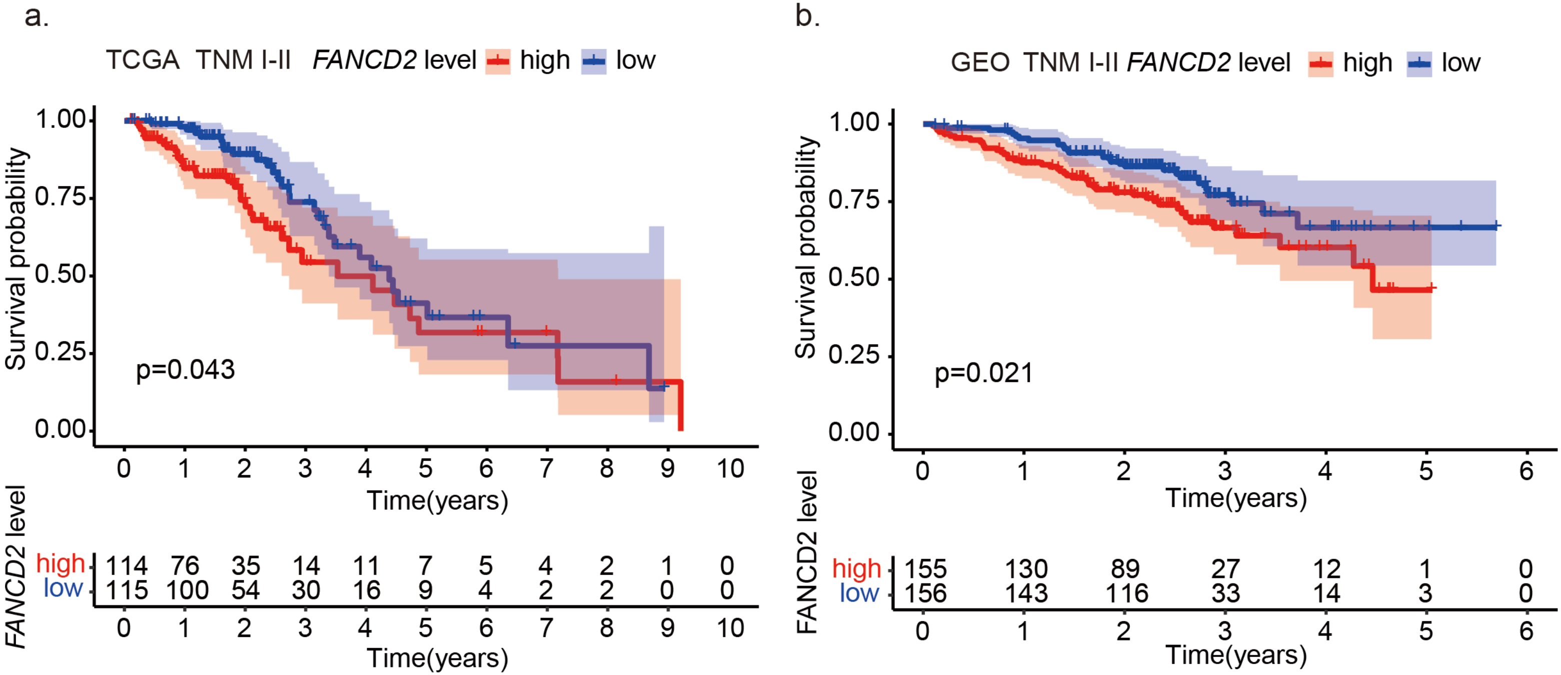

Supplementary Figure 2. Kaplan-Meier survival analysis of *FANCD2* in TCGA and GEO LUAD cohort in TNM I-II.

(a) The high expression of *FANCD2* is related to poor survival in the TNM I-II TCGA-LUAD cohort.

(b The high expression of *FANCD2* is associated with poor survival in the TNM I-II GEO-LUAD cohort.
