# Supplementary material for: Comprehensive analysis of the autophagy-dependent ferroptosis-related gene FANCD2 in lung adenocarcinoma
Source: BMC Cancer. 2022 Mar 2;22:225. doi: 10.1186/s12885-022-09314-9 (PMC8889748; doi:10.1186/s12885-022-09314-9)
Supplement: Supplementary file 3 — Additional file 3. [file 12885_2022_9314_MOESM3_ESM.pdf]

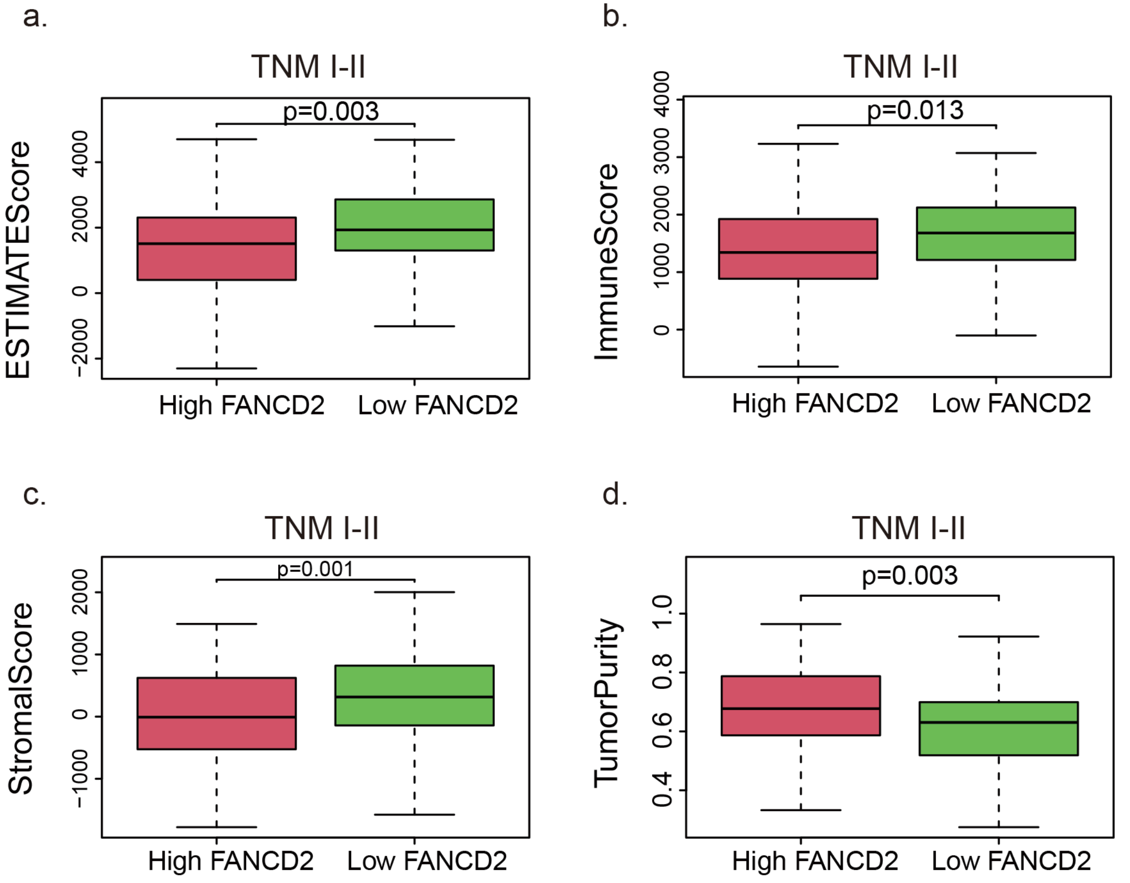

Supplementary Figure 3. The correlation between FANCD2 and ESTIMATE Score (a), Immune Score (b), Stromal Score (c), Tumor Purity (d) in TNM I-II stage TCGA LUAD cohort.
