# Supplementary material for: Comprehensive analysis of the autophagy-dependent ferroptosis-related gene FANCD2 in lung adenocarcinoma
Source: BMC Cancer. 2022 Mar 2;22:225. doi: 10.1186/s12885-022-09314-9 (PMC8889748; doi:10.1186/s12885-022-09314-9)
Supplement: Supplementary file 4 — Additional file 4. [file 12885_2022_9314_MOESM4_ESM.pdf]

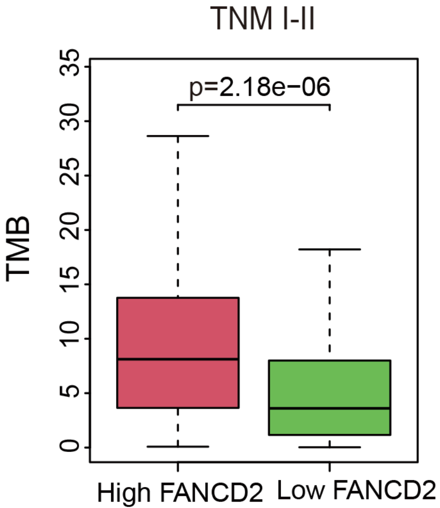

Supplementary Figure 4. The correlation between FANCD2 and TMB in the TNM I-II stage TCGA-LUAD cohort.
