# Supplementary material for: Comprehensive analysis of the autophagy-dependent ferroptosis-related gene FANCD2 in lung adenocarcinoma
Source: BMC Cancer. 2022 Mar 2;22:225. doi: 10.1186/s12885-022-09314-9 (PMC8889748; doi:10.1186/s12885-022-09314-9)
Supplement: Supplementary file 5 — Additional file 5. [file 12885_2022_9314_MOESM5_ESM.pdf]

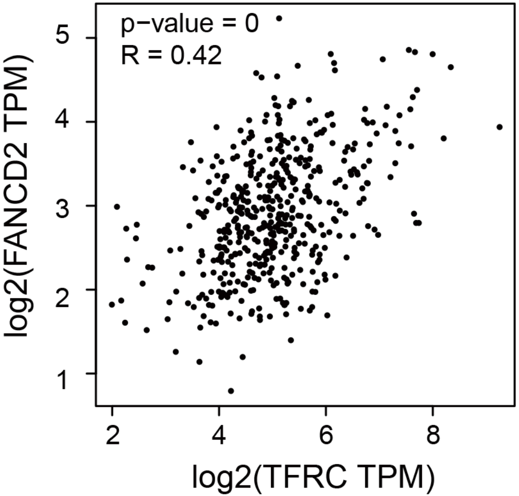

Supplementary Figure 5. The correlation analysis of FANCD2 with ferroptosis marker TFRC in GEPIA database. TFRC: transferrin receptor.
