# Supplementary material for: Comprehensive analysis of the autophagy-dependent ferroptosis-related gene FANCD2 in lung adenocarcinoma
Source: BMC Cancer. 2022 Mar 2;22:225. doi: 10.1186/s12885-022-09314-9 (PMC8889748; doi:10.1186/s12885-022-09314-9)
Supplement: Supplementary file 6 — Additional file 6. [file 12885_2022_9314_MOESM6_ESM.docx]

**Supplementary Table 1. The different expressed genes between LUAD and normal lung tissues.**

| **Gene** | **conMean** | **LUAD-Mean** | **logFC** | **pValue** | **FDR** |
| --- | --- | --- | --- | --- | --- |
| *ALOX15* | 9.771 | 2.355 | -2.052 | 4.25E-12 | 1.01E-11 |
| *ALOX5* | 46.254 | 14.173 | -1.706 | 4.96E-27 | 8.81E-26 |
| *CBS* | 0.068 | 0.192 | 1.484 | 0.000322 | 0.000423 |
| *CHAC1* | 1.363 | 2.800 | 1.037 | 2.61E-12 | 6.40E-12 |
| *DPP4* | 10.721 | 29.928 | 1.481 | 0.000545 | 0.000704 |
| *FANCD2* | 0.637 | 2.093 | 1.715 | 5.56E-28 | 1.97E-26 |
| *GCLC* | 2.798 | 14.655 | 2.388 | 1.11E-11 | 2.40E-11 |

logFC: log2 foldchange; FDR: false discovery rate;
