# Supplementary material for: Comprehensive analysis of the autophagy-dependent ferroptosis-related gene FANCD2 in lung adenocarcinoma
Source: BMC Cancer. 2022 Mar 2;22:225. doi: 10.1186/s12885-022-09314-9 (PMC8889748; doi:10.1186/s12885-022-09314-9)
Supplement: Supplementary file 7 — Additional file 7. [file 12885_2022_9314_MOESM7_ESM.docx]

**Supplementary Table 2. The prognostic genes in TCGA-LUAD cohort.**

| **Gene** | **HR** | **HR.95CI** | **pValue** |
| --- | --- | --- | --- |
| *TMEM173* | 0.775 | 0.657-0.916 | 0.003 |
| *PEBP1* | 0.691 | 0.516-0.925 | 0.013 |
| *KRAS* | 1.351 | 1.066-1.711 | 0.013 |
| *FANCD2* | 1.420 | 1.056-1.910 | 0.020 |
| *COPZ1* | 1.685 | 1.137-2.498 | 0.009 |
| *CISD1* | 1.650 | 1.196-2.276 | 0.002 |
| *ARNTL* | 0.680 | 0.480-0.963 | 0.030 |
| *AGER* | 0.877 | 0.805-0.955 | 0.003 |

HR: Hazard Ratio; HR.95CI: Hazard Ratio 95% Confidence Interval.
