# Supplementary material for: Comprehensive analysis of the autophagy-dependent ferroptosis-related gene FANCD2 in lung adenocarcinoma
Source: BMC Cancer. 2022 Mar 2;22:225. doi: 10.1186/s12885-022-09314-9 (PMC8889748; doi:10.1186/s12885-022-09314-9)
Supplement: Supplementary file 8 — Additional file 8. [file 12885_2022_9314_MOESM8_ESM.docx]

**Supplementary Table 3. Propensity score matching of the low and high *FANCD2* group in TCGA-LUAD dataset.**

| **Variables** | **Low *FANCD2*** | **High *FANCD2*** | **Standardized diff.** | **P value** |
| --- | --- | --- | --- | --- |
| **Age** | 65.36 ± 10.07 | 63.96 ± 10.31 | 0.1372 | 0.199 |
| **Gender** |  | | 0.2070 | 0.068 |
| Male | 69 (39.2) | 87 (49.4) |  | |
| Female | 107 (60.8) | 89 (50.6) |  |  |
| **TNM** |  | | | 0. 105 |
| Ⅰ | 116 (65.9) | 93 (52.8) | 0.2685 |  |
| Ⅱ | 27 (15.3) | 43 (24.4) | 0.2293 |  |
| Ⅲ | 28 (15.9) | 26 (14.8) | 0.0315 |  |
| Ⅳ | 5 (2.8) | 14 (8) | 0.2278 |  |
| **Tumor** |  | | | 0.138 |
| 1 | 76 (43.2) | 56 (31.8) | 0.2364 |  |
| 2 | 80 (45.5) | 101 (57.4) | 0.2405 |  |
| 3 | 12 (6.8) | 11 (6.2) | 0.0230 |  |
| 4 | 8 (4.5) | 8 (4.5) | 0.0000 |  |
| **Node** |  | | | 0.206 |
| 0 | 130 (73.9) | 114 (64.8) | 0.1981 |  |
| 1 | 25 (14.2) | 37 (21) | 0.1797 |  |
| 2 | 21 (11.9) | 24 (13.6) | 0.0511 |  |
| 3 | 0 (0) | 1 (0.6) | 0.1069 |  |
| **Metastasis** |  | | 0.1982 | 0.086 |
| 0 | 147 (83.5) | 133 (75.6) |  | |
| 1 | 29 (16.5) | 43 (24.4) |  |  |

LUAD, adenocarcinoma of lung; TCGA, The Cancer Genome Atlas; Standardized diff.: standardized difference.
