# Supplementary material for: Comprehensive analysis of the autophagy-dependent ferroptosis-related gene FANCD2 in lung adenocarcinoma
Source: BMC Cancer. 2022 Mar 2;22:225. doi: 10.1186/s12885-022-09314-9 (PMC8889748; doi:10.1186/s12885-022-09314-9)
Supplement: Supplementary file 9 — Additional file 9. [file 12885_2022_9314_MOESM9_ESM.docx]

**Supplementary Table 4. Propensity score matching of the low and high *FANCD2* group in GEO-LUAD dataset.**

| **Variables** | **Low *FANCD2*** | **High *FANCD2*** | **Standardized diff.** | **P value** |
| --- | --- | --- | --- | --- |
| **Age** | 69.40 ± 9.44 | 68.73 ± 9.49 | 0.0704 | 0.508 |
| **TNM** |  | | | 0.606 |
| Ⅰ | 105 (59.3) | 116 (65.5) | 0.1286 |  |
| Ⅱ | 35 (19.8) | 29 (16.4) | 0.0882 |  |
| Ⅲ | 31 (17.5) | 25 (14.1) | 0.0930 |  |
| Ⅳ | 6 (3.4) | 7 (4) | 0.0300 |  |
| **Gender** |  | | 0.0692 | 0.588 |
| Male | 109 (61.6) | 103 (58.2) |  | |
| Female | 68 (38.4) | 74 (41.8) |  |  |
| ***TP53*** |  | | 0.1213 | 0.311 |
| Wild | 132 (74.6) | 141 (79.7) |  | |
| Mutant | 45 (25.4) | 36 (20.3) |  |  |

Standardized diff.: standardized difference.
