# Supplementary material for: Comprehensive analysis of the autophagy-dependent ferroptosis-related gene FANCD2 in lung adenocarcinoma
Source: BMC Cancer. 2022 Mar 2;22:225. doi: 10.1186/s12885-022-09314-9 (PMC8889748; doi:10.1186/s12885-022-09314-9)
Supplement: Supplementary file 10 — Additional file 10. [file 12885_2022_9314_MOESM10_ESM.docx]

**Supplementary Table 5. Coefficients of *FANCD2* and Stage variables in TCGA-LUAD cohort.**

| **Model** | | **Unstandardized Coefficients** | | **Standardized Coefficients** | **t** | **Sig.** | **Collinearity Statistics** | |
| --- | --- | --- | --- | --- | --- | --- | --- | --- |
|  |  | **B** | **Std.Error** | **Beta** |  |  | **Tolerance** | **VIF** |
| 1 | (Constant) | 3.009 | 0.396 |  | 7.595 | 0.000 |  |  |
|  | *FANCD2* | -0.325 | 0.199 | -0.094 | -1.634 | 0.103 | 0.986 | 1.014 |
|  | Stage | -0.382 | 0.223 | -0.098 | -1.714 | 0.088 | 0.986 | 1.014 |

Sig: significance; VIF : Variance Inflation Factor;
