# Supplementary material for: Comprehensive analysis of the autophagy-dependent ferroptosis-related gene FANCD2 in lung adenocarcinoma
Source: BMC Cancer. 2022 Mar 2;22:225. doi: 10.1186/s12885-022-09314-9 (PMC8889748; doi:10.1186/s12885-022-09314-9)
Supplement: Supplementary file 11 — Additional file 11. [file 12885_2022_9314_MOESM11_ESM.docx]

**Supplementary Table 6. Collinearity diagnostics of *FANCD2* and Stage variables in TCGA-LUAD cohort**

| **Model** | **Dimension** | **Eigenvalue** | **Condition Index** | **Variance Proportions** | | |
| --- | --- | --- | --- | --- | --- | --- |
|  |  |  |  | **(Constant)** | ***FANCD2*** | **Stage** |
| 1 | 1 | 2.873 | 1.000 | 0.01 | 0.01 | 0.01 |
|  | 2 | 0.089 | 5.689 | 0 | 0.47 | 0.65 |
|  | 3 | 0.039 | 8.634 | 0.99 | 0.52 | 0.34 |
