# Supplementary material for: Comprehensive analysis of the autophagy-dependent ferroptosis-related gene FANCD2 in lung adenocarcinoma
Source: BMC Cancer. 2022 Mar 2;22:225. doi: 10.1186/s12885-022-09314-9 (PMC8889748; doi:10.1186/s12885-022-09314-9)
Supplement: Supplementary file 12 — Additional file 12. [file 12885_2022_9314_MOESM12_ESM.docx]

**Supplementary Table 7. DEGs between the high and low expression level of *FANCD2* in TCGA-LUAD cohort**

| **ID** | **logFC** |
| --- | --- |
| *MFAP4* | -70.4287 |
| *SUSD2* | -58.9371 |
| *RNASE1* | -365.05 |
| *SFTPD* | -259.983 |
| *SFTPB* | -2227.26 |
| *A2M* | -156.78 |
| *CYP4B1* | -56.919 |
| *NAPSA* | -357.052 |
| *C16orf89* | -130.953 |
| *RGCC* | -68.5563 |
| *FOLR1* | -94.5532 |
| *SFTPC* | -1846.95 |
| *MGP* | -57.1909 |
| *EPAS1* | -69.3263 |
| *AGER* | -246.235 |
| *TUBB* | 75.73196 |
| *CST3* | -51.4529 |
| *CTSH* | -89.6158 |
| *DUSP1* | -120.19 |
| *CTSD* | -191.902 |
| *SFTPA1* | -1647.6 |
| *GAPDH* | 325.8428 |
| *SFTA2* | -134.558 |
| *CLDN18* | -65.7276 |
| *HMGA1* | 73.60338 |
| *TPI1* | 67.71922 |
| *LDHA* | 51.28037 |
| *CAV1* | -64.9905 |
| *HLA-DRB1* | -328.667 |
| *HLA-E* | -120.739 |
| *SFTPA2* | -1762.04 |
| *IGFBP7* | -56.3011 |
| *CD63* | -93.8173 |
| *HSP90AA1* | 59.70482 |
| *HLA-DPB1* | -71.1544 |
| *CD74* | -509.808 |
| *ZFP36* | -102.393 |
| *ENO1* | 130.4702 |
| *DHCR24* | -52.2057 |
| *GRN* | -50.9105 |
| *PRDX5* | -79.348 |
| *IGFBP4* | -62.9715 |
| *FOS* | -80.2437 |
| *TXNIP* | -76.4723 |
| *GPX3* | -87.7337 |
| *TMSB4X* | -342.343 |
| *HSP90AB1* | 103.2177 |
| *TPT1* | -133.383 |
| *ATP5F1B* | 51.66391 |
| *HLA-DRA* | -494.475 |
| *SCGB1A1* | -702.656 |
| *EGR1* | -52.3872 |
| *HBB* | -136.43 |
| *ALDOA* | 58.14467 |
| *AQP1* | -108.226 |
| *AQP3* | -97.6657 |
| *VIM* | -52.041 |
| *SLC34A2* | -181.481 |
| *PPDPF* | -61.7848 |
| *NPC2* | -94.9948 |
| *LDHB* | 68.11845 |
| *RHOB* | -50.0207 |
| *HSP90B1* | 51.46916 |
| *HLA-DRB5* | -138.283 |
| *EEF1A1* | -156.068 |
| *WFDC2* | -108.745 |
| *SCGB3A2* | -365.839 |
| *PABPC1* | 75.16754 |
| *C4BPA* | -71.4142 |
| *SLPI* | -707.718 |
| *MUC1* | -56.1297 |
| *MSLN* | -127.601 |
| *KRT18* | 69.30766 |
| *KRT8* | 55.9274 |
| *MT-ND6* | -522.692 |
| *PGC* | -982.395 |
| *MIR3609* | 50.2389 |
| *C1QA* | -52.6282 |
| *RNU4-2* | 460.532 |
| *PRDX1* | 54.18099 |
